# Supplementary material for: Recognition and coacervation of G-quadruplexes by a multifunctional disordered region in RECQ4 helicase
Source: Nat Commun. 2023 Oct 24;14:6751. doi: 10.1038/s41467-023-42503-z (PMC10598209; doi:10.1038/s41467-023-42503-z)
Supplement: Supplementary file 1 — Supplementary Information [file 41467_2023_42503_MOESM1_ESM.pdf]

Supplementary Figure 1

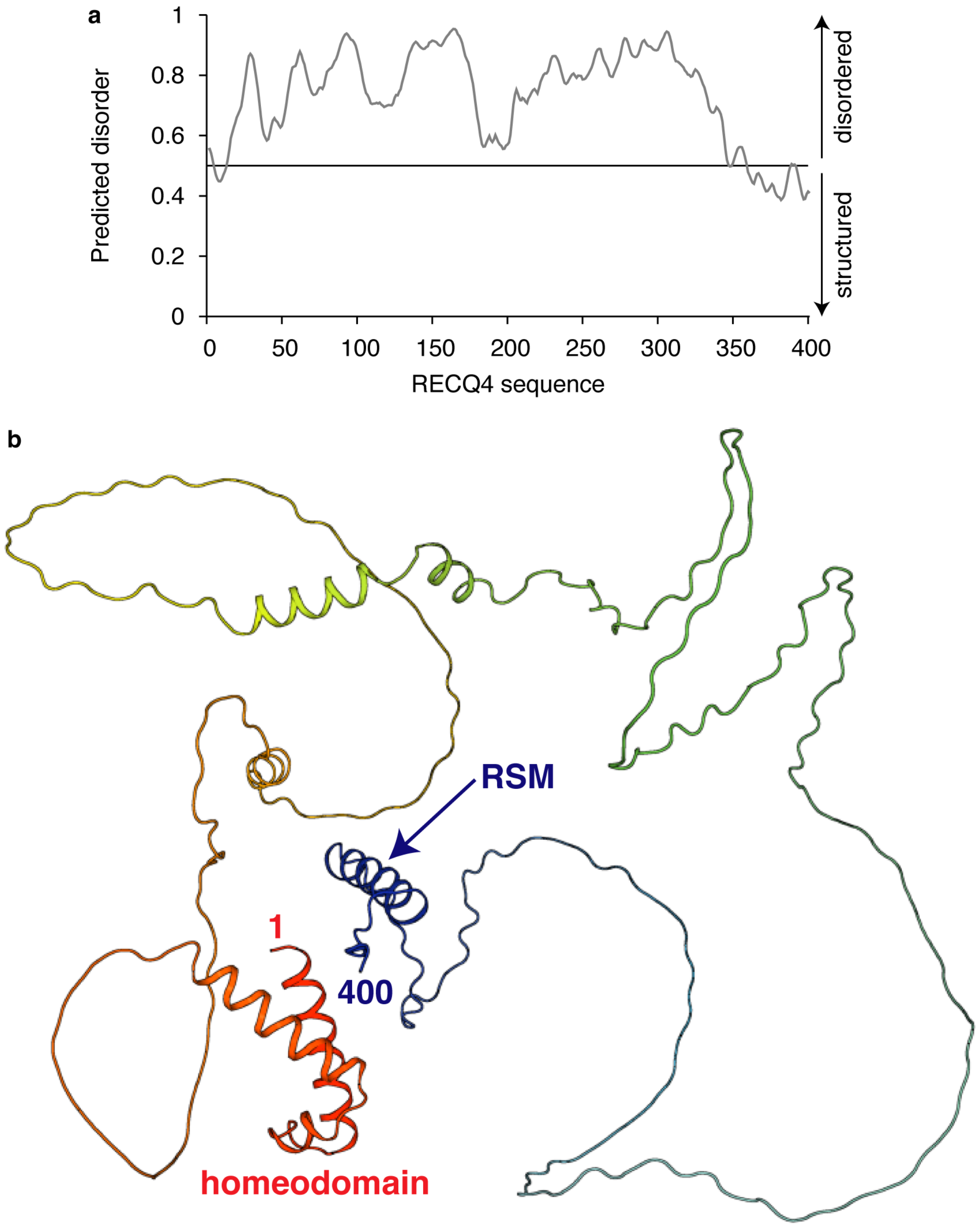

Supplementary Figure 1. The Sld2-like region of RECQ4 is largely disordered. (**a**) Sequence-based prediction of protein disorder for the Sld2-like region of RECQ4 (aa 1-400) using the IUPred3 method [1]. (**b**) Prediction of protein structure using the AlphaFold2 method [2]. Structured homeodomain and RSM region are depicted. Colour coding: red to blue from N- to C-terminus.

Supplementary Figure 2

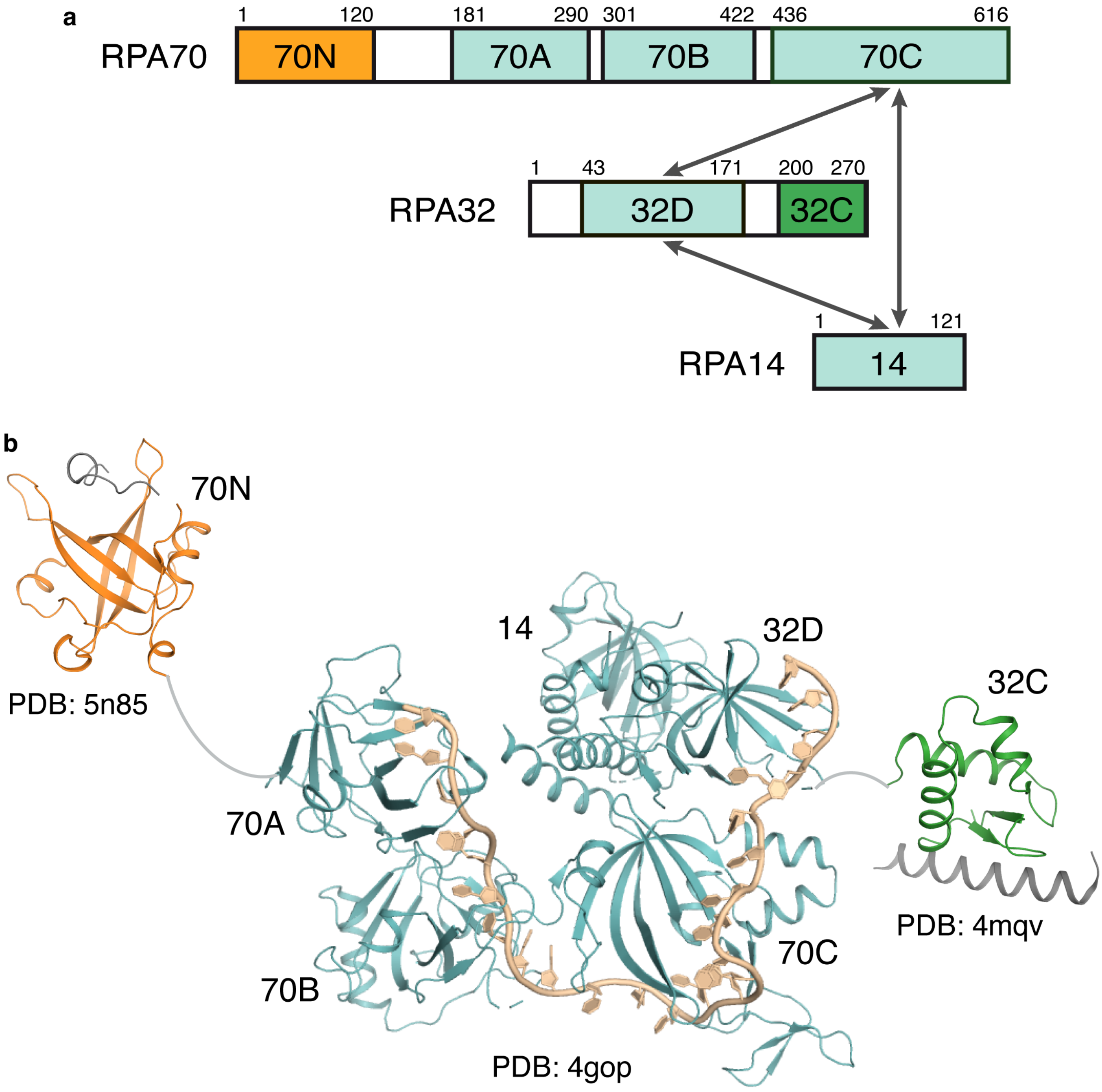

Supplementary Figure 2. Subunit and domain organization of RPA heterotrimer. (**a**) Schematic of human RPA70, RPA32, and RPA14 subunits that form the RPA heterotrimer. The various domains are labeled and the arrows indicate the intersubunit trimeric association. (**b**) Structural model of the RPA modular architecture. The larger subunit RPA70, possesses three DNA binding domains (70A, 70B, 70C; cyan) and a domain that serves as a site of protein–protein interactions (70N; orange). RPA32 subunit is made of a DNA binding domain (32D; cyan) and a WH domain (32C; green) which participates in protein–protein interactions. The smallest subunit RPA14 consist of a single domain (14; cyan) that trimerizes with 70C and 32C domains. 4gop: RPA in complex with ssDNA; 5n85: RPA in complex with PrimPol; 4mqv: RPA in complex with SMARCAL1. Grey lines represent linkers between the domains not present in PDBs.

Supplementary Figure 3

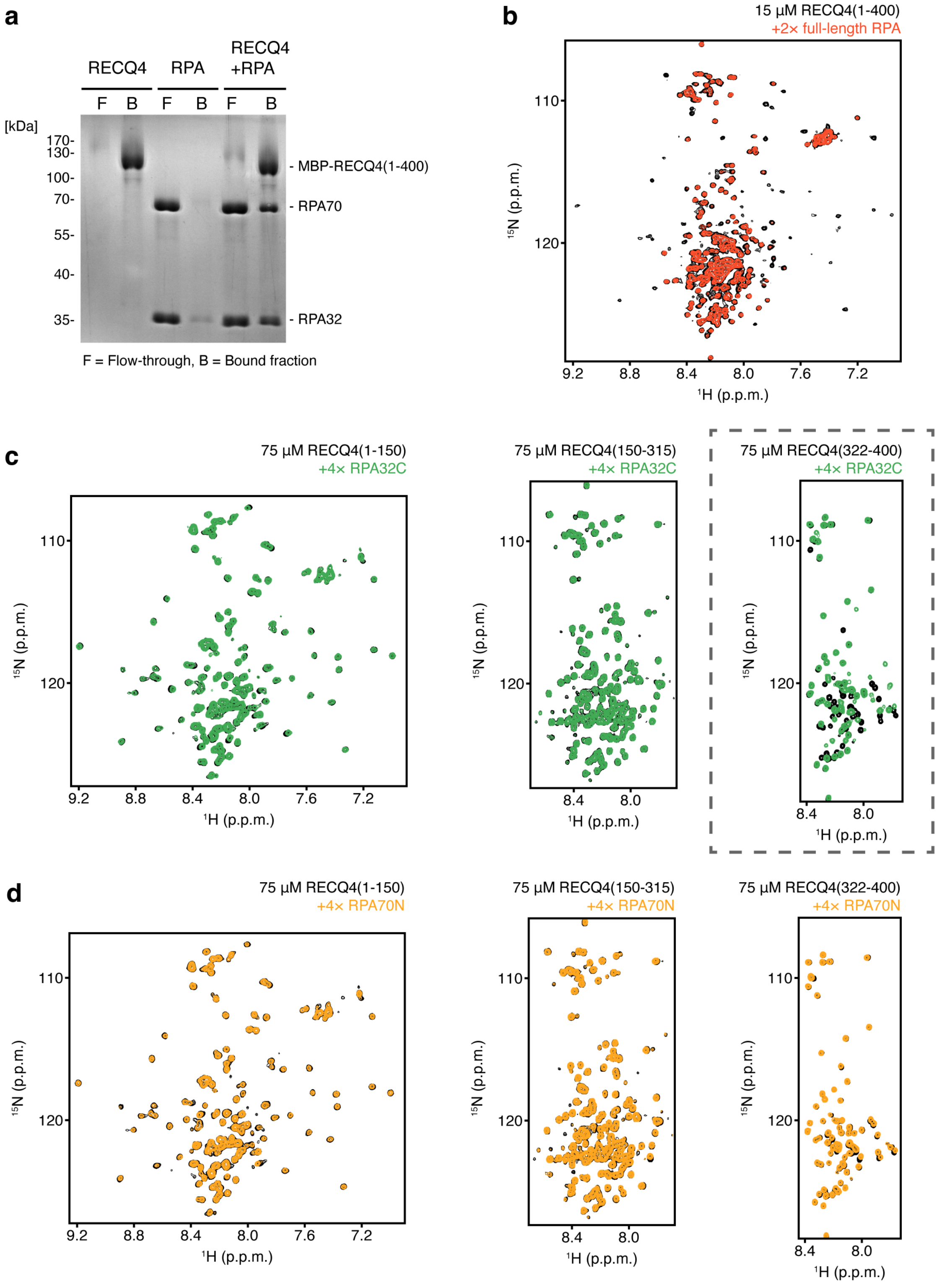

Supplementary Figure 3. Physical interaction between RECQ4 and RPA proteins. (**a**) *In vitro* pull-down assay with recombinant MBP-RECQ4(1-400) and RPA heterotrimer (8 µM each). Proteins were incubated (individually or in combination) with amylose beads and the beads (bound fraction, B) were separated from the flow-through (flow-through fraction, F). Both fractions were analyzed by SDS-PAGE and Coomassie staining. RPA14 is not visible due to its low molecular mass. (**b**) Overlay of ^{1}H-^{15}N HSQC spectra of 15 µM free ^{15}N-RECQ4 (aa 1-400; black) with addition of 2× unlabeled full-length RPA heterotrimer (red). Several peaks broaden beyond detection indicating a physical interaction. (**c**,**d**) Overlay of ^{1}H-^{15}N HSQC spectra of 75 µM free ^{15}N-RECQ4 segments (1-150, 150-315, 322-400; black) with addition of 4× unlabeled RPA32C domain (green, **c**) or 4× unlabeled RPA70N domain (orange, **d**). Specific chemical shift perturbations were observed for the RECQ4(322-400)-RPA32C binary interaction only (dashed grey box). Source data are provided as a Source Data file.

Supplementary Figure 4

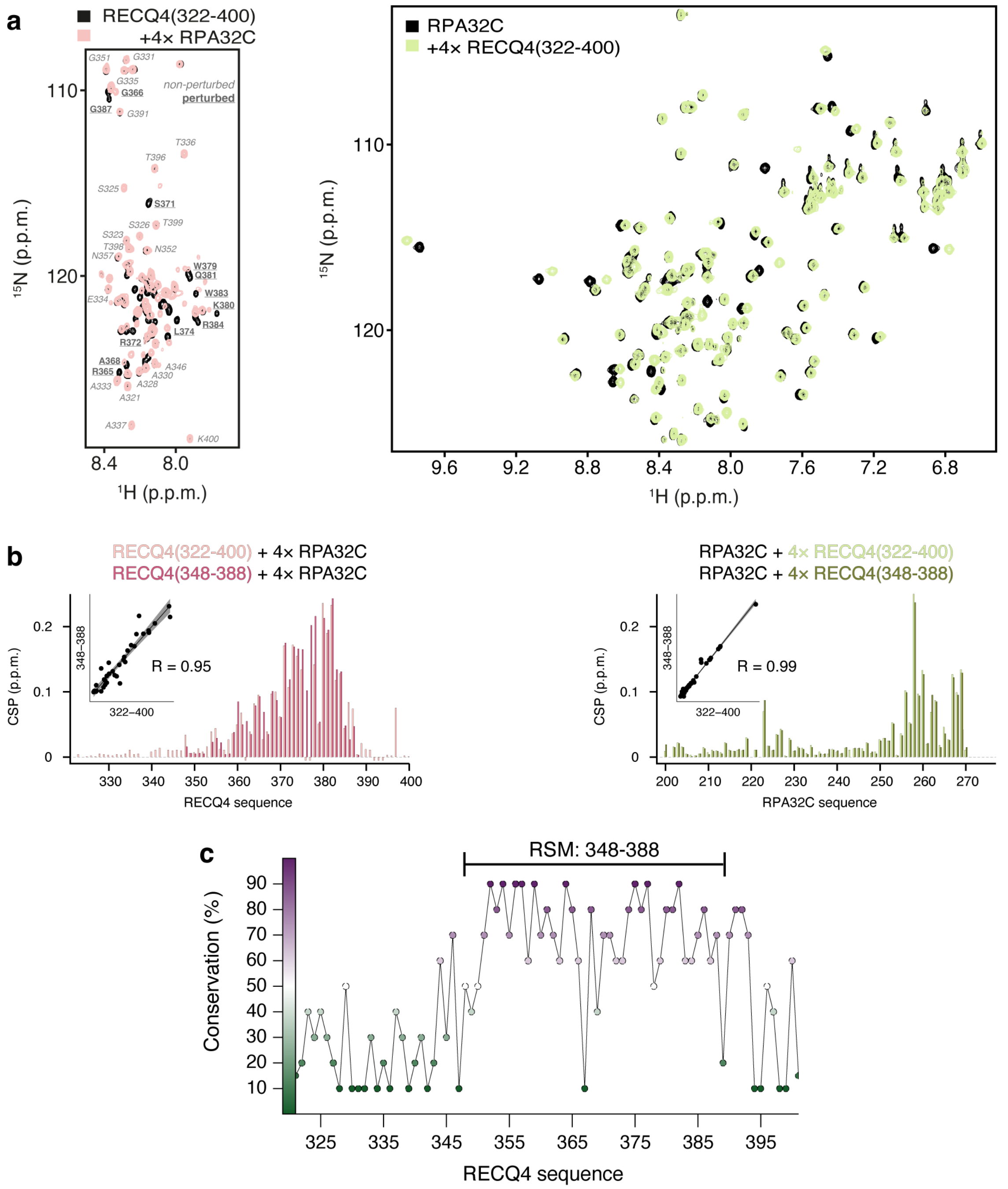

Supplementary Figure 4. A RECQ4-specific motif (RSM; residues 348-388) suffices for the interaction with RPA32C. (**a**) (left) ^{1}H-^{15}N HSQC spectrum of 100 µM free ^{15}N-RECQ4(322-400) and in the presence of 4× molar excess of unlabeled RPA32C. Perturbed (underline) and non-perturbed (italic) residues are highlighted. (right) ^{1}H-^{15}N HSQC spectrum of 100 µM free ^{15}N-RPA32C and in the presence of 4× molar excess of unlabeled RECQ4(322-400). (**b**) (left) CSPs of 100 µM RECQ4(322-400) and of 100 µM RSM segments induced by 4× molar addition of RPA32C and their correlation. (right) CSPs of RPA32C domain induced by 4× molar addition of either RECQ4(322-400) or RSM and their correlation. (**c**) Conservation score from multiple sequence alignment of RECQ4(322-400) using 28 RECQ4 protein sequences was calculated using the Consurf server [3]. The alignment of 28 sequences from UniProt covering the Euteleostomi taxonomic range was generated by MUSCLE1 v3.8.3 [4]. Sequence alignment is available in the Source Data file.

Supplementary Figure 5

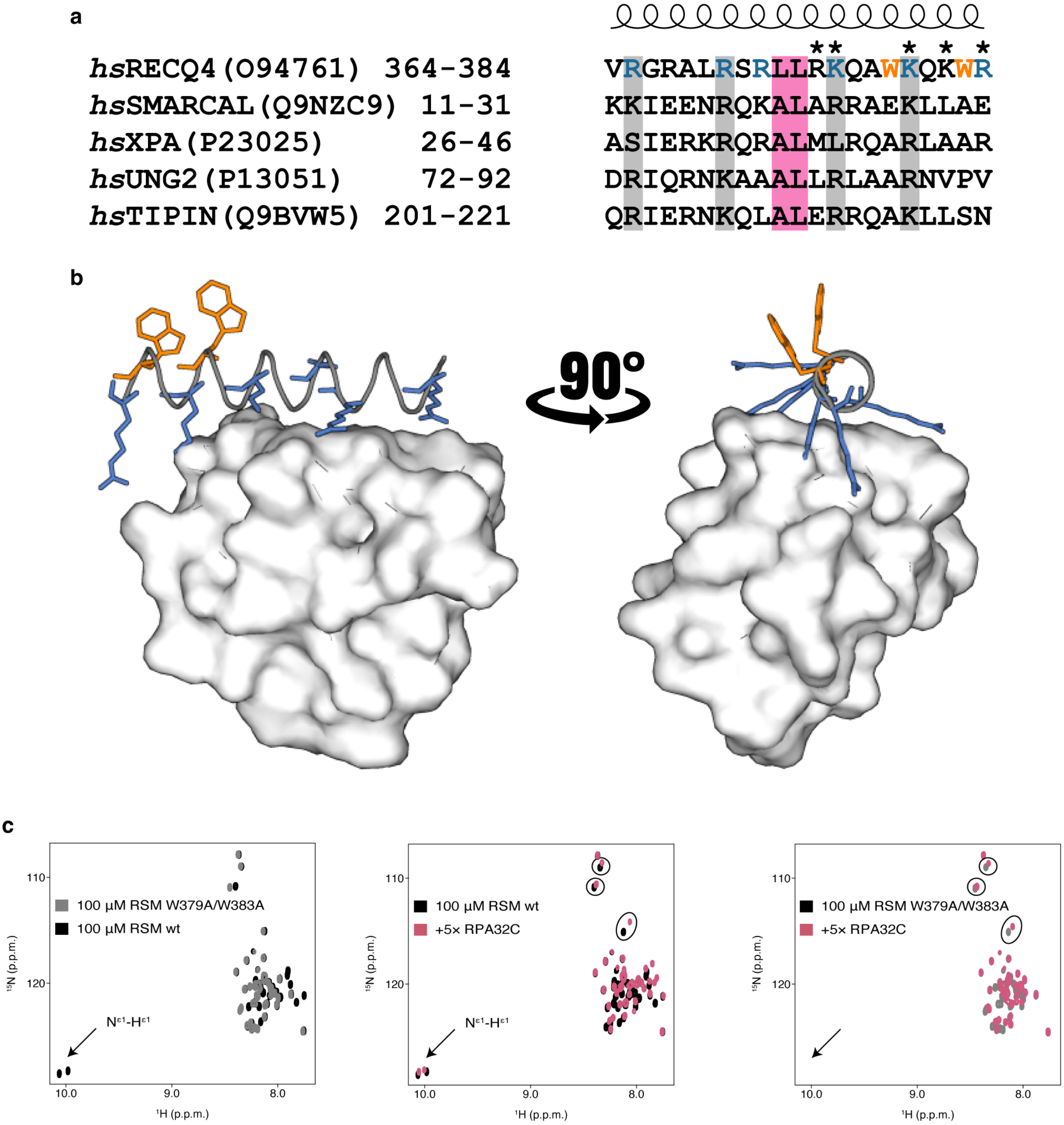

Supplementary Figure 5. Role of electrostatics in RSM-RPA32C interaction. (**a**) Sequence alignment of peptide segments from different proteins known to interact with RPA32C in an induced helix conformation. Positions that retain a positively charged character are highlighted in grey, the conserved hydrophobic dipeptide is highlighted in magenta, and the tryptophan residues of RSM are colored orange. Asterisks above the RECQ4 sequence denote the charge reversal mutations in RSM 5E-mutant. (**b**) A model of RSM-RPA32C interaction based in RPA32C-SMARCAL crystal structure (PDB: 4mqv). RSM positively charged residues in direct contact with RPA32C domain are shown in blue sticks and RSM tryptophan residues projecting away from the binding cleft are shown in orange. (**c**) Overlay of ^{1}H-^{15}N HSQC spectra of 100 μM RSM (WT or W379A/W383A mutant) in the free form and in the presence of 5× molar excess of RPA32C. The peak perturbations of well-resolved peaks are circled. Notice that the magnitude and direction of perturbations are very similar, suggesting that the tryprophans are not critical for RPA32C binding, in accordance with the model in (**b**).

Supplementary Figure 6

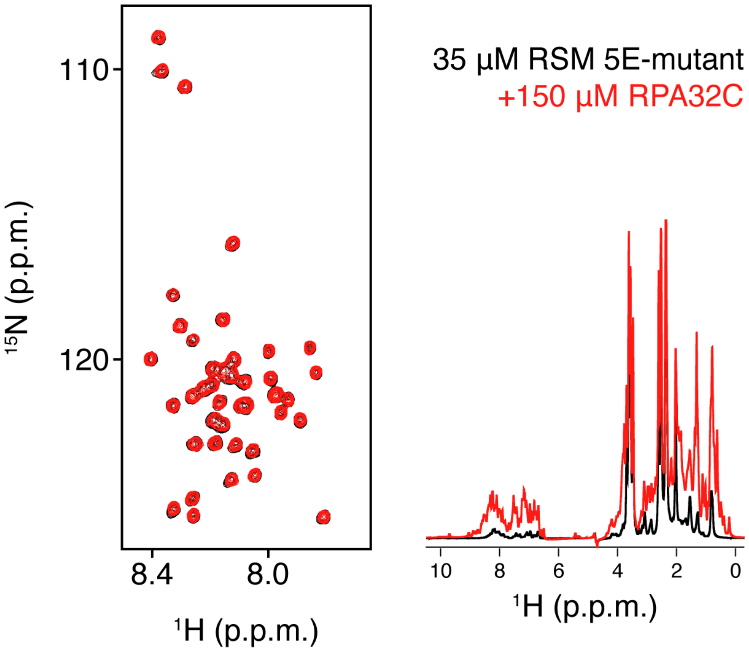

Supplementary Figure 6. The RSM 5E-mutant does not interact with RPA32C. ^{1}H-^{15}N HSQC and 1D ^{1}H NMR spectra of 35 µM free ^{15}N-RSM 5E-mutant (black) and in the presence of 150 µM unlabeled RPA32C (red).

Supplementary Figure 7

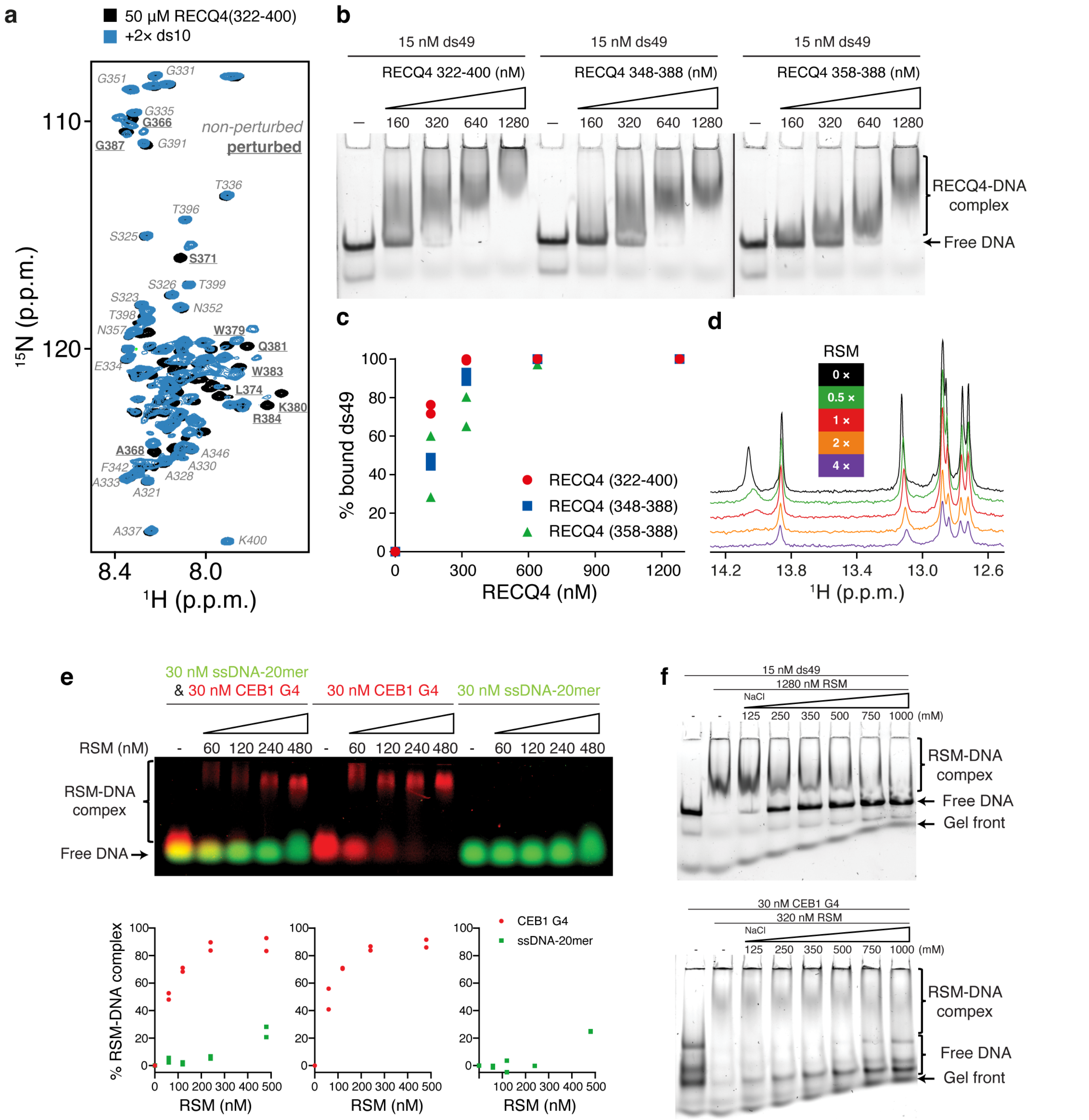

Supplementary Figure 7. RSM interaction with DNA substrates. (**a**) Overlay of ^{1}H,^{15}N HSQC spectra of 50 µM free ^{15}N-RECQ4(322-400) (black) and with 2× molar excess of 10mer dsDNA (ds10; blue). Perturbed (underline) and non-perturbed (italic) residues are highlighted. Only residues within the RSM segment participate in DNA binding. (**b**) EMSAs with 15 nM fluorescently labeled 49mer dsDNA (ds49) and increasing amounts of either RECQ4(322-400), RSM (aa 348-388), or sRSM (aa 358-388). (**c**) Quantification of gels shown in (**b**), n=2 independent experiments. (**d**) Monitoring the chemical shift perturbations of the well-resolved DNA imino protons of 50 µM double-stranded DNA (ds10) with increasing concentrations of RSM. RSM binding does not affect the integrity of the DNA helix. (**e**) Increasing concentration of RSM was incubated with 30 nM CEB1 G4 (red), 30 nM ssDNA-20mer (green) or their equimolar mixture (30 nM each) to assemble RSM-DNA complexes, which were analysed on agarose gel (top). Gel quantification (bottom), n=2 independent experiments. (**f**) EMSA of RSM binding to 15 nM fluorescently labeled 49mer dsDNA (ds49; top) or 30 nM parallel G4 (CEB1; bottom) as a function of ionic strength. Source data are provided as a Source Data file.

Supplementary Figure 8

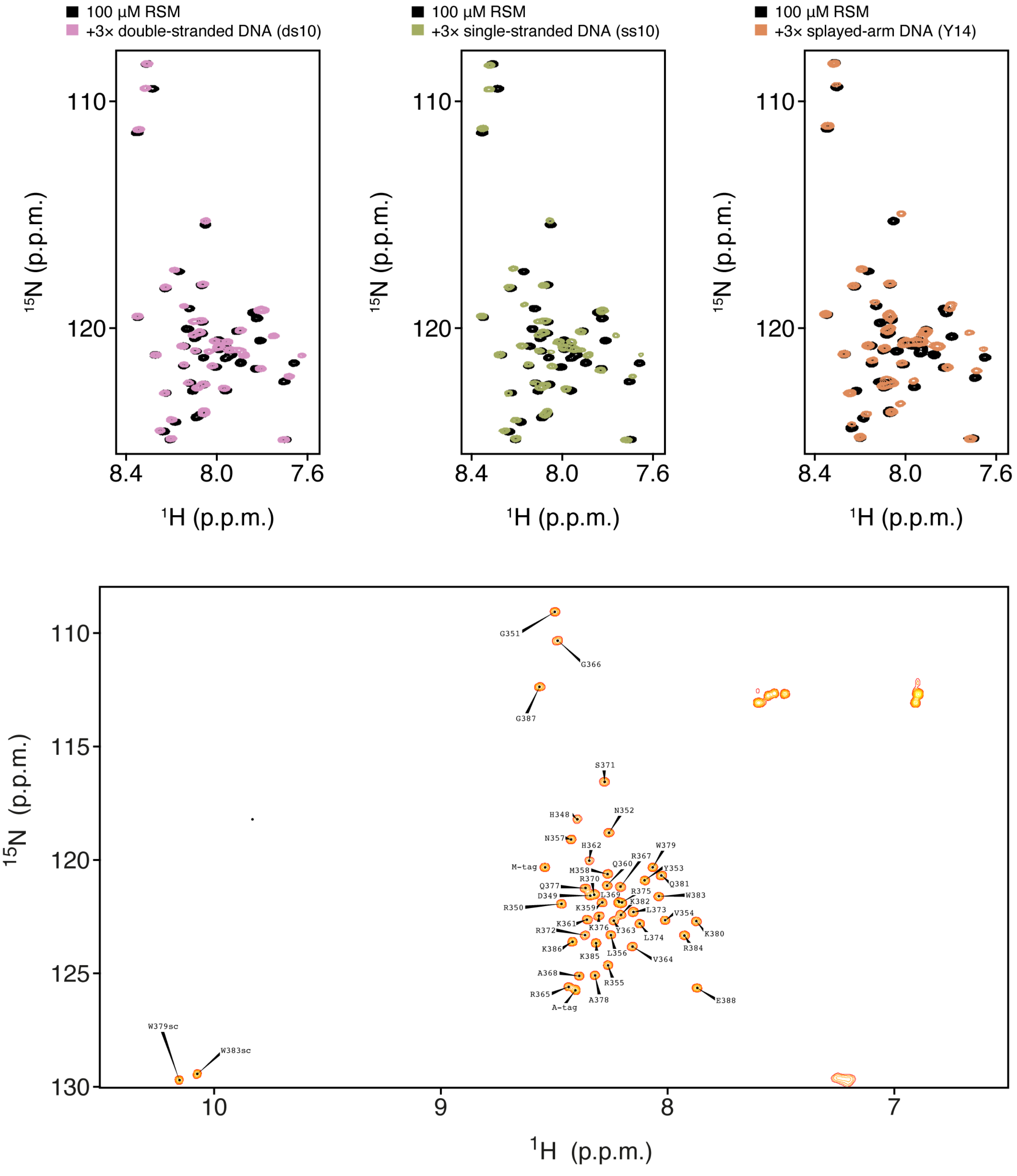

Supplementary Figure 8. RSM binding to various DNA structures monitored by NMR. (top) Overlay of ^{1}H-^{15}N HSQC spectra of 100 µM free ^{15}N-RSM (black) and in the presence of 3× molar excess of either double-stranded (ds10; purple), single-stranded (ss10; green), or splayed-arm (Y14; orange) DNA. (bottom) Full ^{1}H-^{15}N HSQC spectrum of RSM. Peak assignemnts are indicated according to the RECQ4 sequence.

Supplementary Figure 9

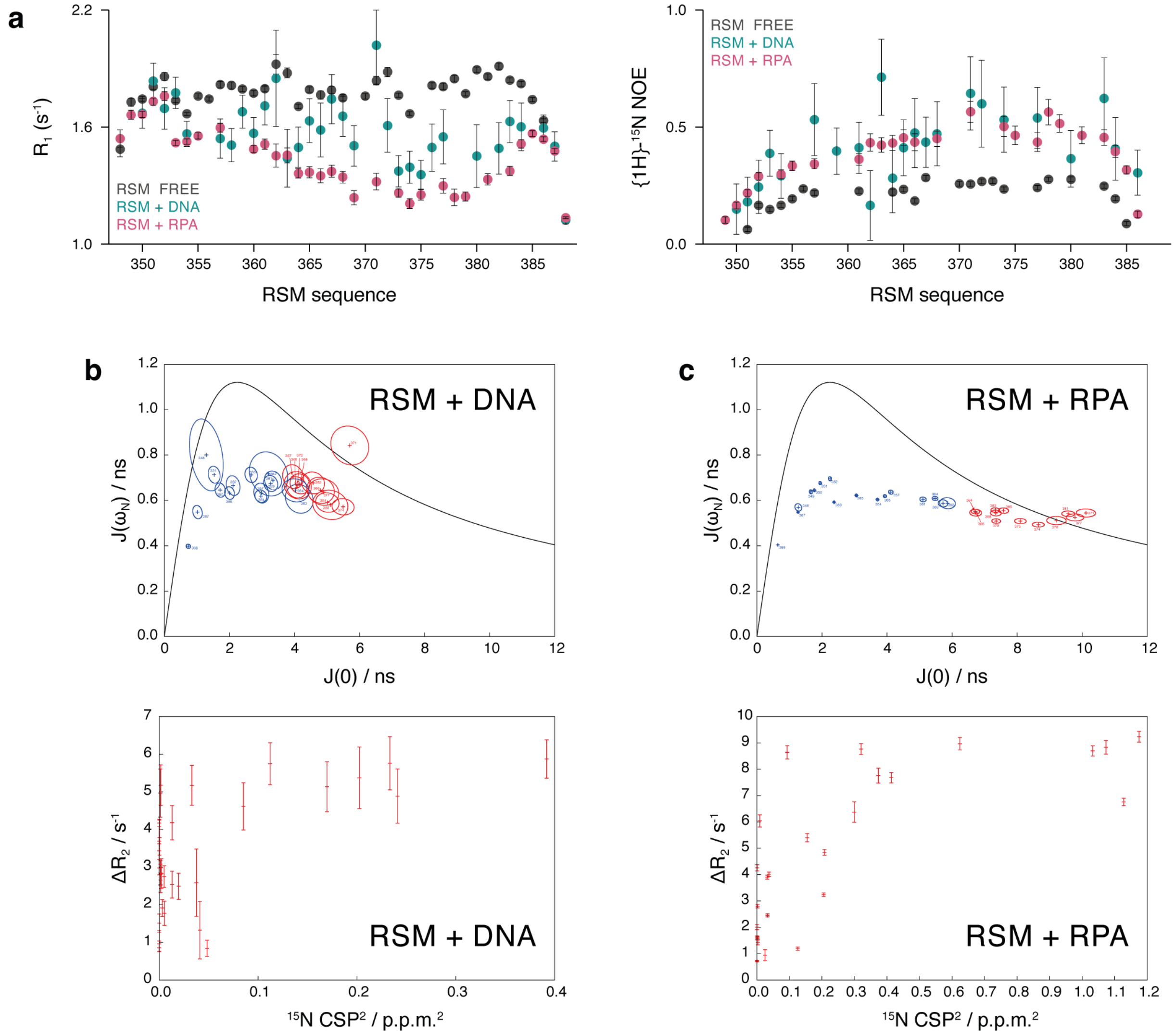

Supplementary Figure 9. Characterization of dynamics in free and bound RSM. (**a**) Relaxation parameters (^{15}N R_1 and $\{^1H\}$-^{15}N NOE) measured for free RSM (black; 50 µM RSM) and RSM in complex with DNA (cyan; 4× molar excess 10mer dsDNA) or RPA (pink; 16× molar excess RPA32C). Relaxation and steady-state NOE experiments were performed once. Error bars in relaxation experiments represent the standard error of the fitted parameters. Errorbars in steady-state NOE were calulated using error propagation procedure. (**b**,**c**) Correlation maps of the spectral density function of $J(\omega_N)$ at 700 MHz vs J(0) for RSM in complex with DNA (**b**) or in complex with RPA (**c**). The spectral density function is taken from $J(\omega)=\tau_c/(1+(\omega*\tau_c)^2$, where τ_c is the correlation time and ω is the frequency. The black line represents the theoretical limit for motions described by a single correlation time without exchange contributions. The result for each RSM residue is represented by its position given by the cross and the estimation of the uncertainties depicted by the ellipses. Residues on the right side of the theoretical limit exhibit exchange contributions. To exclude the possibility that R_2 relaxation rates from residues involved in binding are only due to exchange, we plotted the difference of R_2 values as a function of the square of difference of nitrogen frequency between the free and bound states of RSM and found no correlation (bottom). This suggests that the increase of R_2 values is not only attributable to exchange contribution but to changes in motions at ps-ns timescale as well. RSM binding residues (red) show elevated rigidity when in RPA complex as opposed to when in DNA complex. Source data are provided as a Source Data file.

Supplementary Figure 10

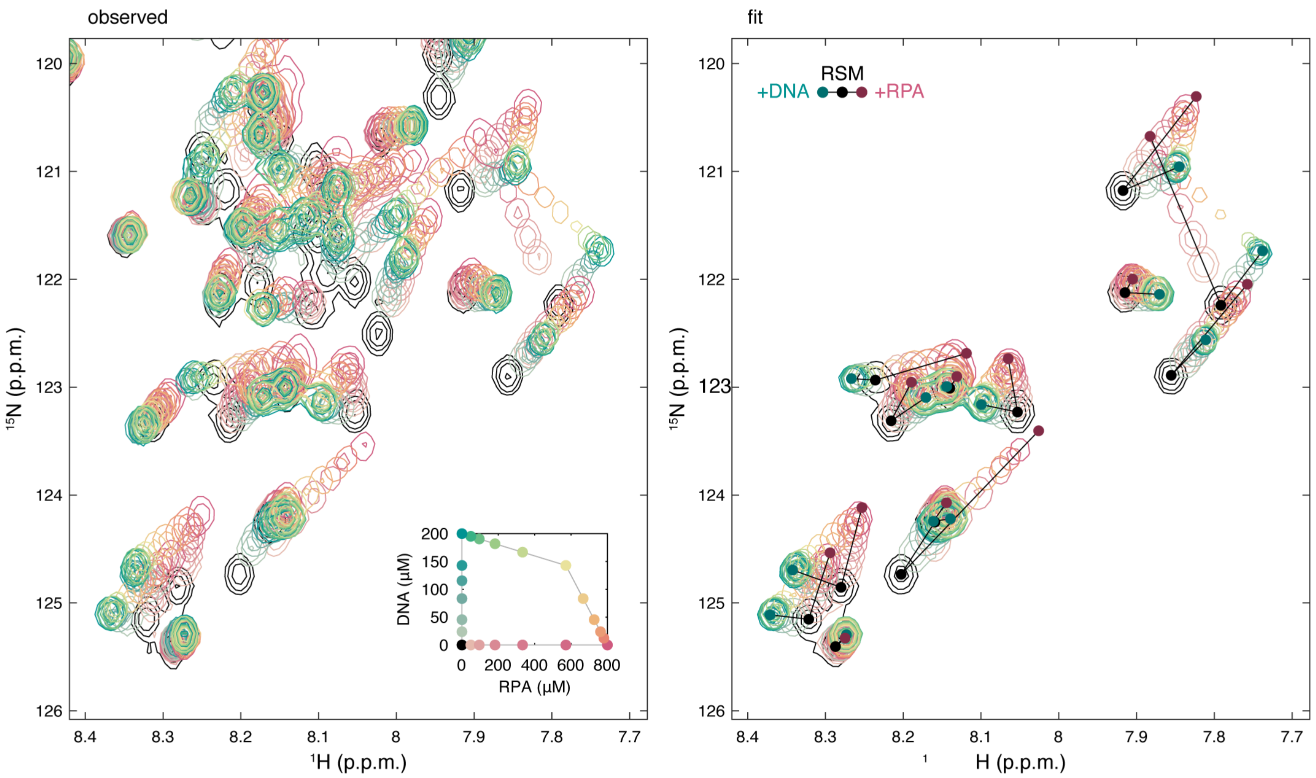

Supplementary Figure 10. Monitoring double-stranded DNA (ds10) and RPA32C competition for RSM binding by NMR. (left) Overlay of 24 titration points monitoring 50 μM RSM (black) upon binding to 4× molar excess of DNA (black to green) and challenged then with increasing amounts of RPA (green to yellow) or upon binding to 16× molar excess of RPA (black to purple) and challenged then with increasing amounts of DNA (purple to yellow). Inset shows DNA and RPA concentrations at different titration points corresponding to peak colours in the overlaid spectra. (right) 2D lineshape analysis of selected peak trajectories for global fitting in TITAN using a competitive binding model.

Supplementary Figure 11

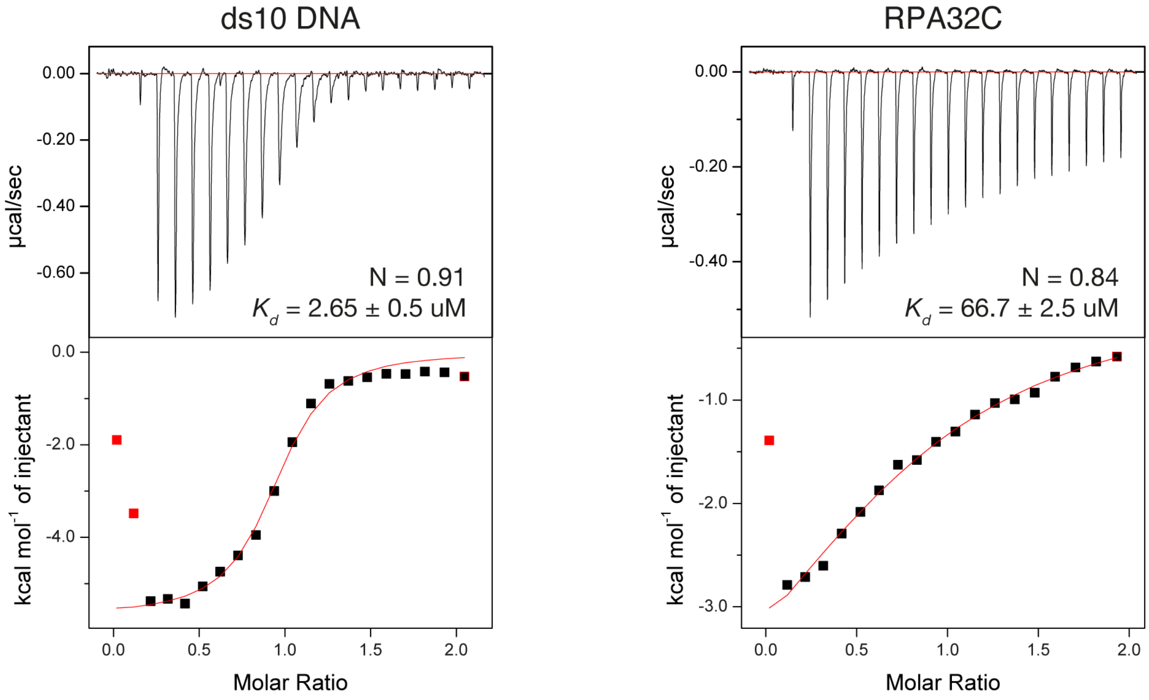

Supplementary Figure 11. ITC thermograms of RSM (100 µM in the cell) titrated with ds10 DNA (1 mM in the syringe) or RPA32C (1 mM in the syringe). The binding affinity and stoichiometry of each interaction are depicted. Red points were excluded from fitting. Source data are provided as a Source Data file.

Supplementary Figure 12

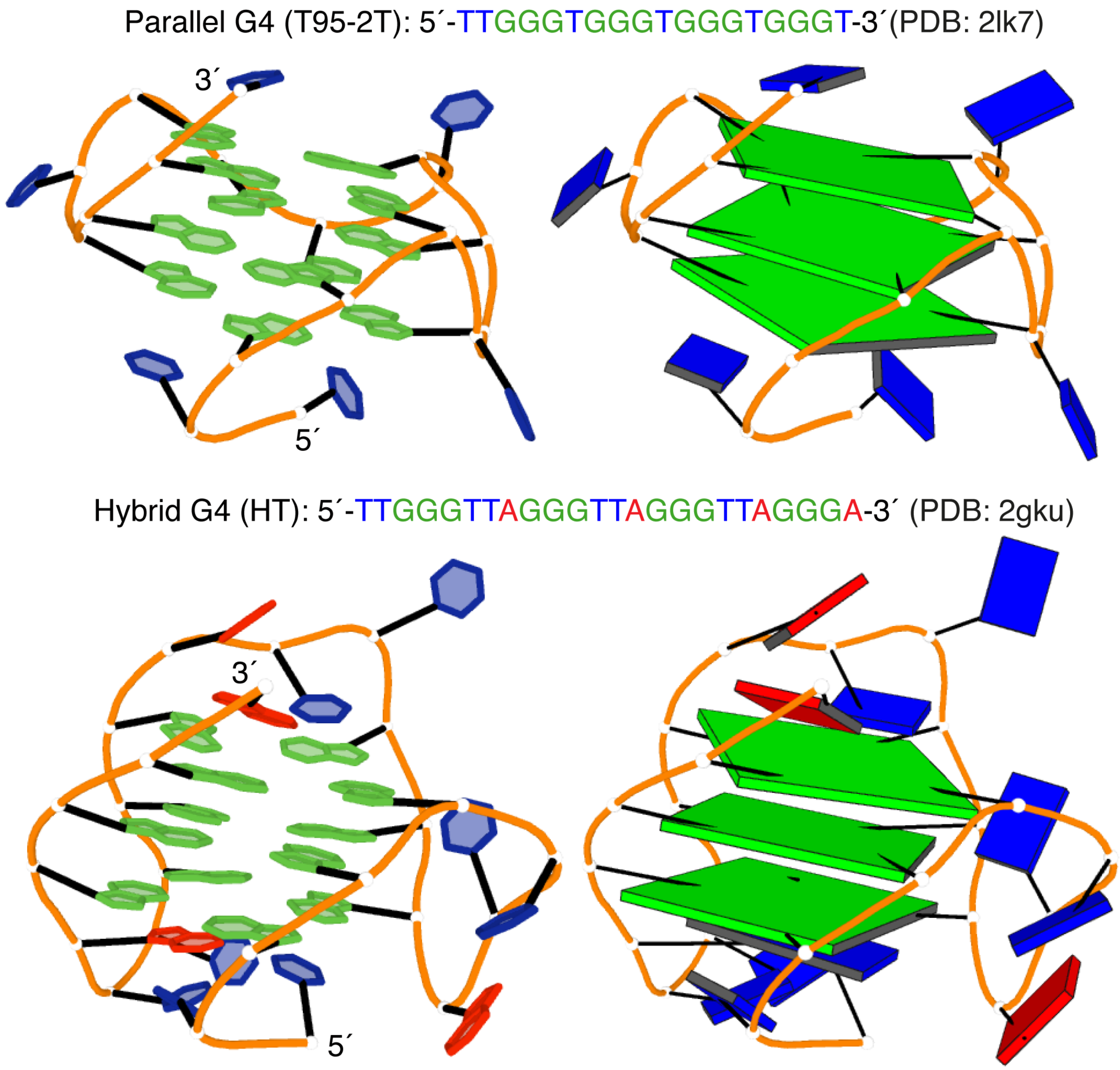

Supplementary Figure 12. G4 structures used in studying RSM-G4 complexes. NMR solution structures of parallel G4 (T95-27, top) and hybrid G4 (HT, bottom). Only the base rings are depicted as sticks (left) or as rectangles (right) to highlight the G-quartet planes. Guanines are shown in green, thymines in blue, and adenines in red.

Supplementary Figure 13

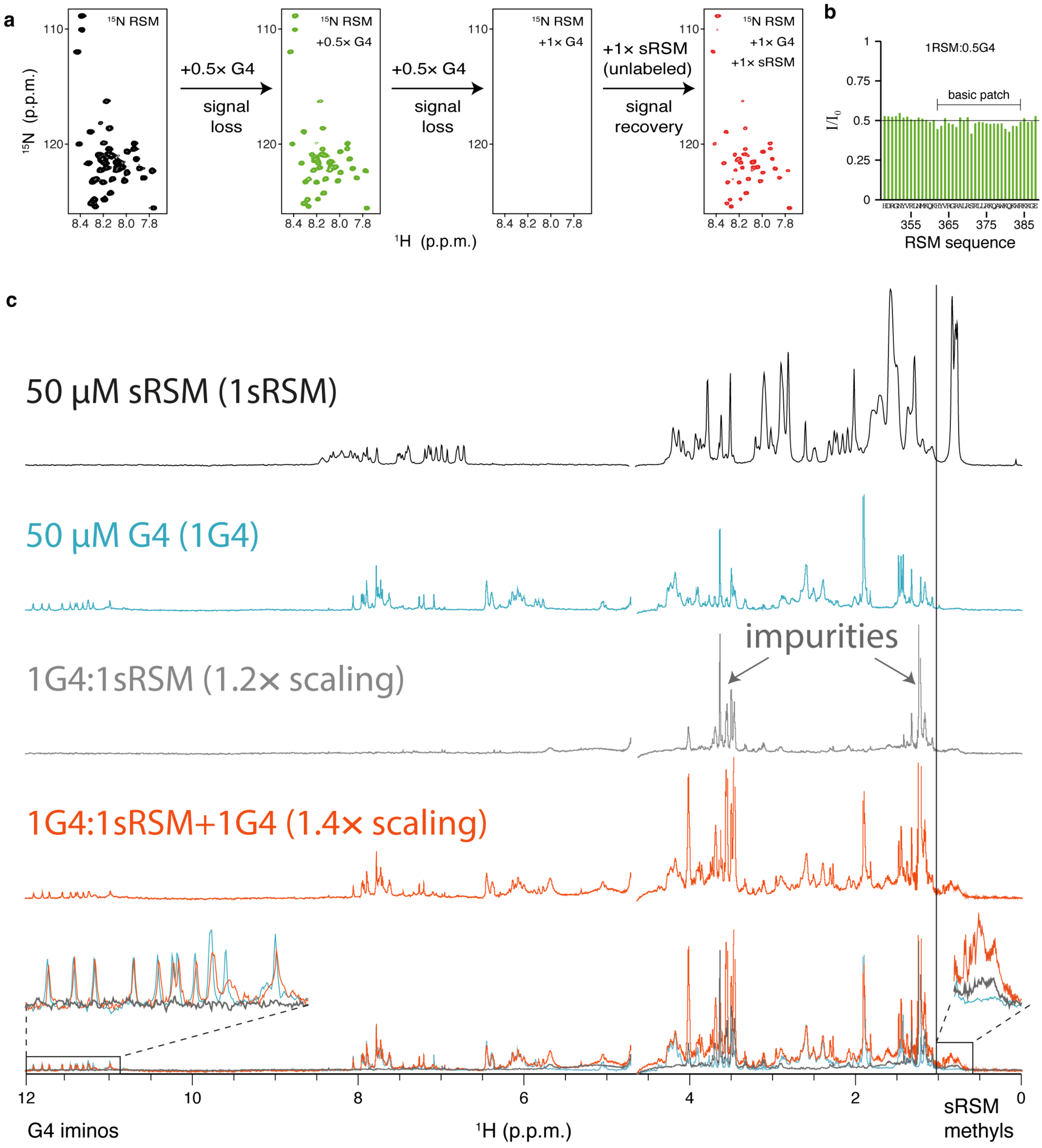

Supplementary Figure 13. RSM-G4 equilibrium between dilute and condensed phases. (**a**) Addition of parallel G4 (T95-2T) to 50 µM ^{15}N labeled RSM results in signal loss due to the formation of phase-separated droplets. Addition of extra unlabeled sRSM redistributes ^{15}N labeled RSM between RSM-G4 complex in condensed phase and free RSM in dilute phase (signal recovery). (**b**) Loss of signal intensity for RSM residues induced by 0.5× molar addition of parallel G4 (T95-2T). Basic patch residues are affected more than the general trend. (**c**) Proton NMR spectra of 50 µM free sRSM (black), 50 µM free T95-2T G4 (cyan), stoichiometric sRSM-G4 complex that partitions in the condensed phase (grey; no signal), and after addition of extra 50 µM G4 (orange) in the phase separated sample. The extra G4 reestablishes the equilibrium between dilute and condensed phases (overlay at the bottom). G4 signals report the weighted average between free and sRSM-bound states in the dilute phase, as evidenced by the recovery of sRSM methyl signals in the NMR spectrum. For proper signal comparison, grey and orange spectra were scaled to account for sample dilution.

Supplementary Figure 14

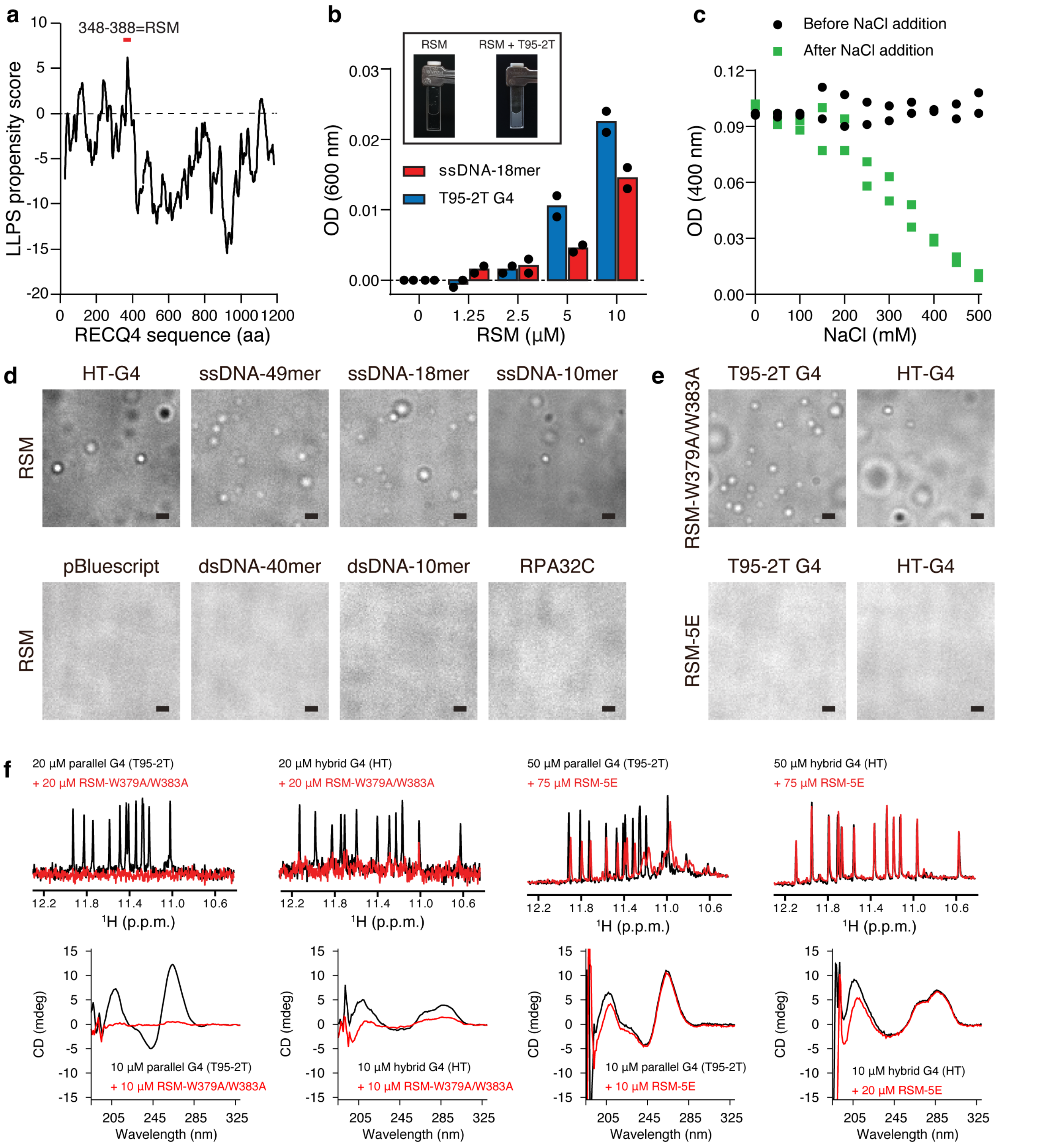

Supplementary Figure 14. Liquid-liquid phase separation (LLPS) of RSM and its mutants. (**a**) RECQ4 sequence-based LLPS propensity calculated using the catGRANULE algorithm [5]. RSM is highlighted above the graph. (**b**) T95-2T G4 or ssDNA-18mer (10 µM each) were mixed with increasing amount of RSM and sample turbidity was measured as light absorbance at 600 nm. The background turbidity was subtracted and average values are shown, n = 2 independent experiments. Inset shows turbidity images of solutions containing 10 µM RSM alone or mixed with equimolar amount of T95-2T G4. (**c**) T95-2T G4 (10 µM) was mixed with sRSM (10 µM). After verification of droplets formation the indicated concentration of NaCl was added.Turbidity was measured as light absorbance at 400 nm. The bacground turbidity was subtracted, n=2 independent experiments. (**d**,**e**) Phase separation microscopy of 10 µM RSM or its mutants (RSM-5E and RSM-W379A/W383A) with equimolar amounts of various DNA substrates or RPA32C protein. In all images scale bar corresponds to 1 µm. (**f**) NMR and CD measurements of respective RSM mutants and their mixtures with parallel (T95-2T) or hybrid (HT) G4s. Source data are provided as a Source Data file.

Supplementary Figure 15

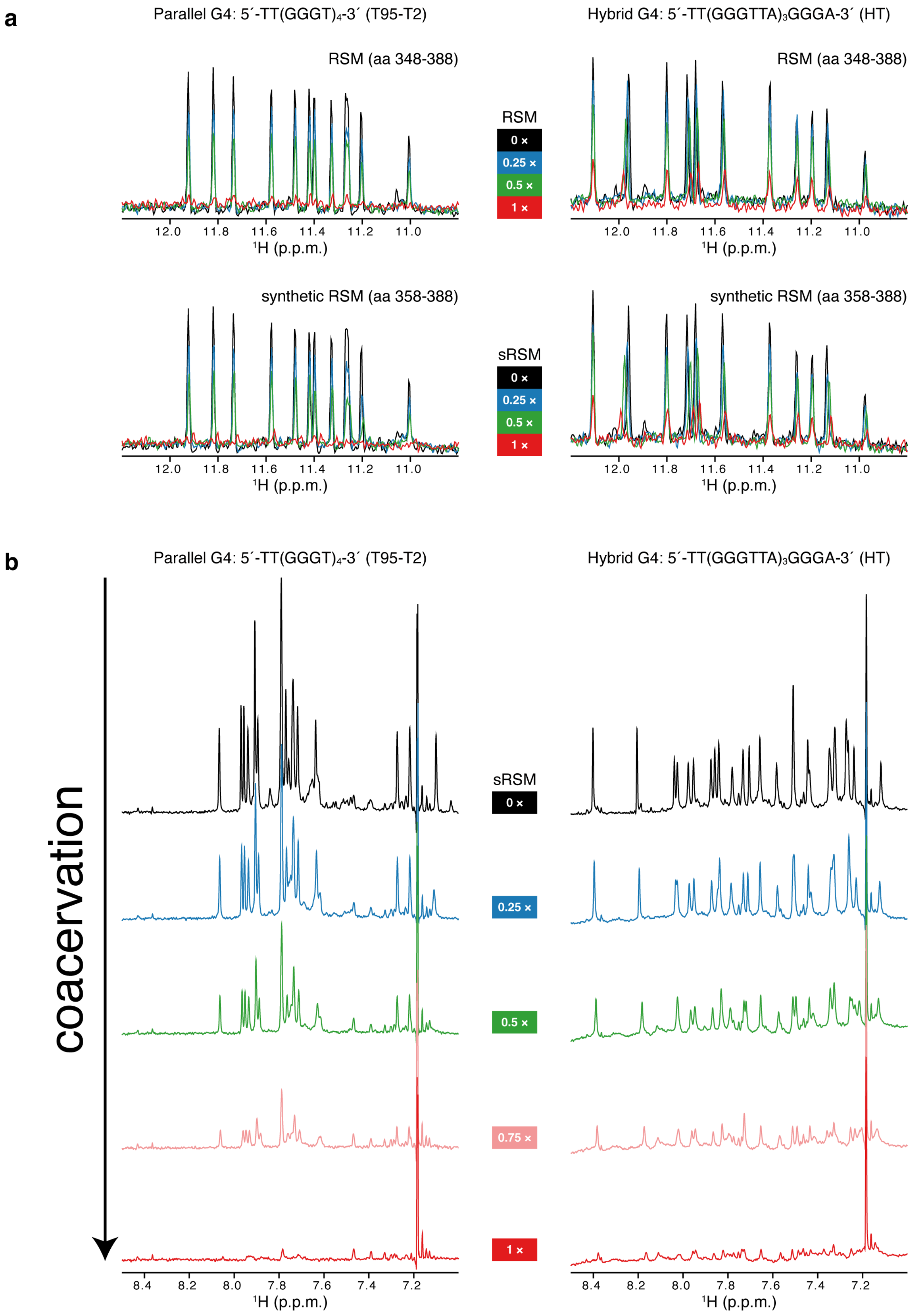

Supplementary Figure 15. Effect of RSM-G4 coacervation on NMR signals. (**a**) Signal loss and CSPs of G4 imino signals (50 µM samples) compared at given stoichiometries of RSM (aa 348-388) or synthetic RSM (aa 358-388). (**b**) G4 aromatic signals decrease in intensity as the G4 coacervated molecules produce no NMR signal due to slow molecular tumbling and fast transverse relaxation of the droplets. Similarly, sRSM amide peaks do not appear as the added sRSM molecules partition to the dense coacervate phase.

Supplementary Figure 16

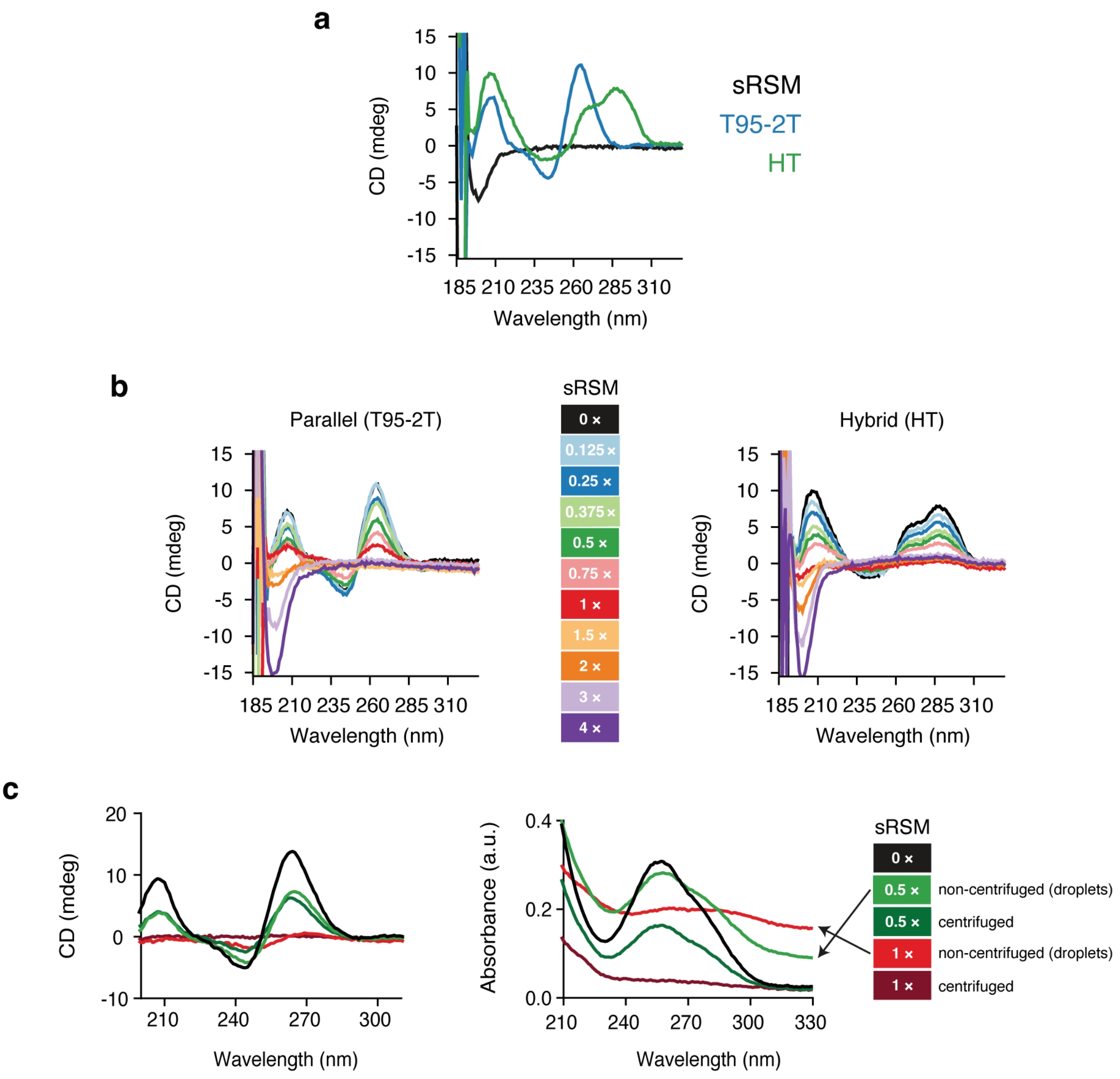

Supplementary Figure 16. CD spectroscopy of sRSM-G4 complexes. (**a**) CD spectra of 10 µM sRSM, 10 µM parallel G4 (T95-2T) or 10 µM hybrid G4 (HT). (**b**) CD spectra of 10 µM G4s (T95-2T and HT) at various G4:sRSM stoichiometries. (**c**) The droplets appear optically inactive. CD (left) and absorbance (right) spectra of 10 µM free T95-2T G4, 1:0.5 T95-2T:sRSM, and 1:1 T95-2T:sRSM with and without centrifugation. The absorption of the non-centrifuged samples is not close to zero at 320-330 nm (where DNA does not absorb), indicative of light scattering due to droplets in the sample. The magnitude of the CD signal is proportional to G4 molecules in the dilute phase (in centrifuged samples phases have been separated). Source data are provided as a Source Data file.

Supplementary Figure 17

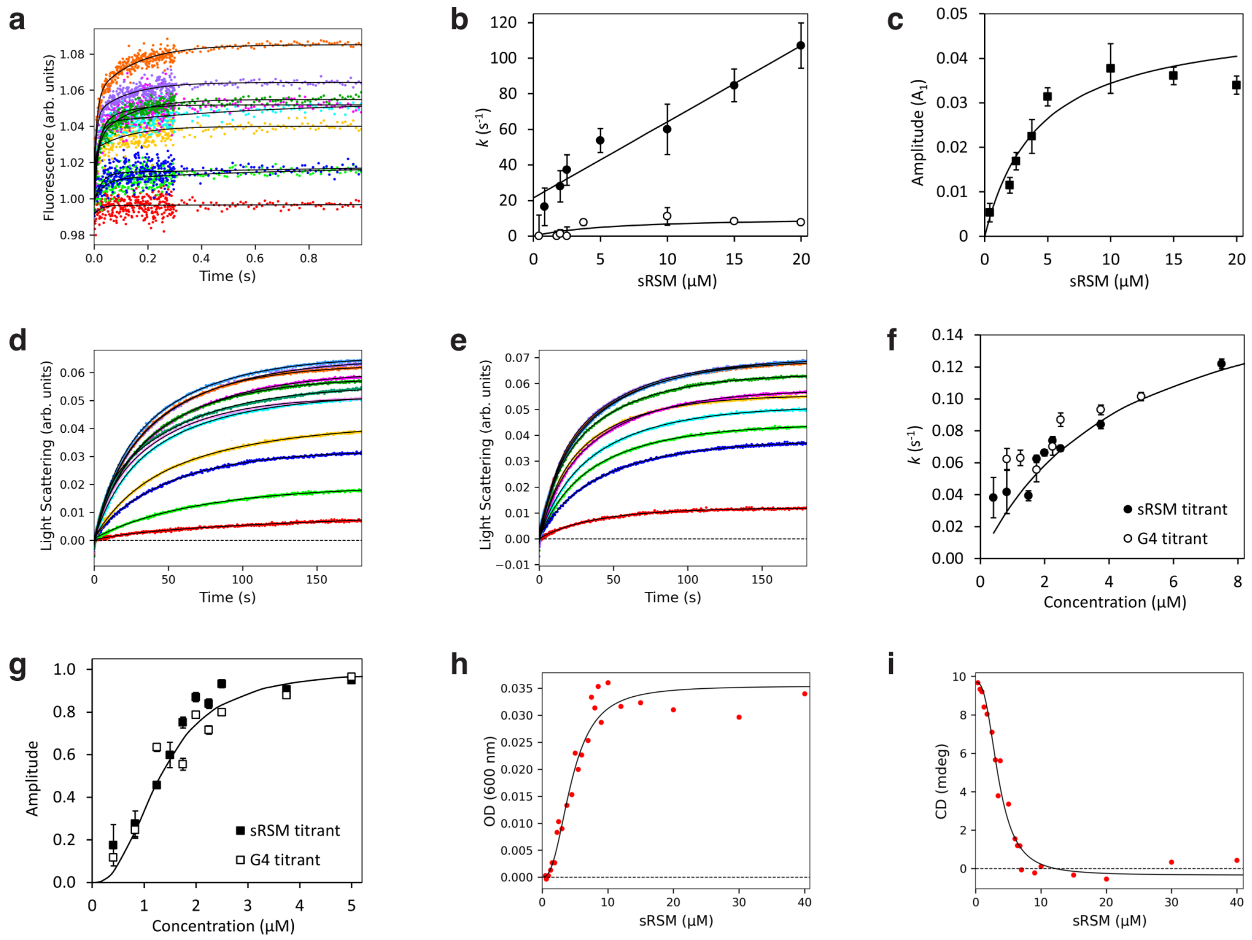

Supplementary Figure 17. Analytical fitting of kinetic data. (**a**) The initial phase of the reaction was examined by stopped-flow fluorescence (excitation 295 nm, emission > 320 nm) upon mixing 2.5 µM G4 with 0 – 20 µM sRSM. The solid lines represent fit to a double exponential function (see equation in **Supplementary Note**). (**b**) The concentration dependence of the rates of the fast and slow phases derived from fitting data in (**a**). The solid lines represent the best fit to the linear model and hyperbola for the fast and slow phases, respectively. (**c**) The concentration dependence of the amplitude of the fast phase (A1). The solid line corresponds to the hyperbolic curve that provides the best fit to the data. (**d**) The kinetic traces recorded using light scattering upon rapid mixing 2.5 µM G4 with 0 – 20 µM sRSM and (**e**) 2.5 µM sRSM with 0 – 20 µM G4. The solid lines represent fit to a double exponential function. (**f**) The concentration dependence of the observed rates derived from fitting data in (**d**) (black circles) and (**e**) (open circles). The solid line corresponds to the hyperbolic curve that provides the best fit to the data. (**g**) The concentration dependence of the total amplitude (at equilibrium) derived from fitting data in (**d**) (black circles) and (**e**) (open circles). The solid line corresponds to the hyperbolic curve that provides the best fit to the data. The error bars in (**b**), (**c**), and (**f**) show the standard errors of the parameters obtained during the initial exponential fit. (**h**) The optical density recorded at 600 nm and (**i**) circular dichroism (CD) spectra recorded upon the titration of 10 µM G4 with a range of 0 to 40 µM sRSM. The solid line corresponds to the Hill model that provides the best fit to the data. In panels (**b**), (**c**), (**f**) and (**g**) error bars represent the standard error of the fitted parameters. Source data are provided as a Source Data file.

Note to Supplementary Figure 17

Conventional analytical fitting of the kinetic data.

We started by fitting stopped-flow data analytically to develop a kinetic model and obtain initial estimates of the rate and equilibrium constants. Natural tryptophan fluorescence of sRSM provided a valuable information on initial steps of sRSM-G4 interaction without any need for labelling. In the first second of the reaction (**Supplementary Fig. 17a**), the fluorescence intensity increased exhibiting two kinetic phases that fit a double exponential function:

$$f = F_0 + A_1(1 - e^{-k_{\mathrm{fast}}t}) + A_2(1 - e^{-k_{\mathrm{slow}}t})$$

The concentration dependence of the rate of the fast phase (k_{fast}) provided the initial estimates of the association k_1 = 4.3 ± 0.4 μM^{-1} s^{-1} and dissociation k_{-1} = 21.5 ± 4.0 s^{-1} rate constants for formation of initial sRSM-G4 binding complex (**Supplementary Fig. 17b**). The value of the equilibrium dissociation constant for the initial sRSM-G4 complex ($K_{d,1}$ = 4.3 ± 0.9 μM) obtained by fitting the concentration dependence of the amplitude (A_1) (**Supplementary Fig. 17c**) corresponds well with the value calculated from rate constants ($K_{d,1} = k_{-1}/k_1$ = 5.0 μM) indicating consistency of the analytical fitting. The second step (k_{slow}) approaches a limiting observed rate of 10 s^{-1}. Under the simplifying assumption that the majority of G4 molecules associates with sRSM after the first step, when the k_1[RSM] >> k_2 + k_{-1}, the forward association velocity is greater than the sum of rates leading to disappearance of the sRSM-G4 complex, we can obtain a rough estimate of the rate constant for the second step (k_2) as 4 ± 2 s^{-1}. The fluorescence intensity provided a satisfactory signal when utilizing sRSM as a titrant in an excess concentration. However, when sRSM was employed as an analyte (during the symmetrical titration of G4 in excess), the low concentration of sRSM did not yield a signal sufficient for achieving good accuracy in the corresponding analysis of G4 titration.

Next, the kinetic measurement was conducted using stopped-flow with signal collection in light scattering mode, allowing to monitor the kinetics of the ultimate formation of condensation products during liquid-liquid phase separation. Unlike the fluorescence intensity, the universal light scattering signal allowed for symmetrical analysis of both the sRSM titration (**Supplementary Fig. 17d**) and the G4 titration (**Supplementary Fig. 17e**). Both sets of data exhibited similar kinetic profiles, suggesting an equivalent role of sRSM and G4 in their mutual association. The observed rate obtained by the analytical fit of light scattering data (**Supplementary Fig. 17f**) equilibrates in an asymptote of 0.19 ± 0.3 s^{-1} providing a rough estimate for the velocity at which the condensation process occurs. The sigmoidal shape of the concentration dependence of the amplitude fitting well to Hill model (n = 2.5 ± 0.7) suggested a “cooperative” mode of sRSM-G4 assembly (**Supplementary Fig. 17g**), where a weak initial binding is followed by further steps with increasing mutual affinity of later association partners. The same sigmoidal shape of the concentration dependence was observed for titration experiments using optical density measurements (**Supplementary Fig. 17h**) and CD (**Supplementary Fig. 17i**) with Hill coefficient n = 2.7 ± 0.3 and 2.5 ± 0.2, respectively. Furthermore, both titration experiments unambiguously demonstrated a 1:1 stoichiometry in the sRSM-G4 assembly.

Analytical fitting offers valuable insights into the mechanism, particularly through the examination of rate and amplitude dependencies on concentration. However, it is limited in providing precise parameter estimates due to approximations and the accumulation of errors during multistep fitting. To address these limitations, we finally employed the global modeling approach by numerically integrating the rate equations derived from the proposed kinetic model to analyze the kinetic data comprehensively.

Supplementary Figure 18

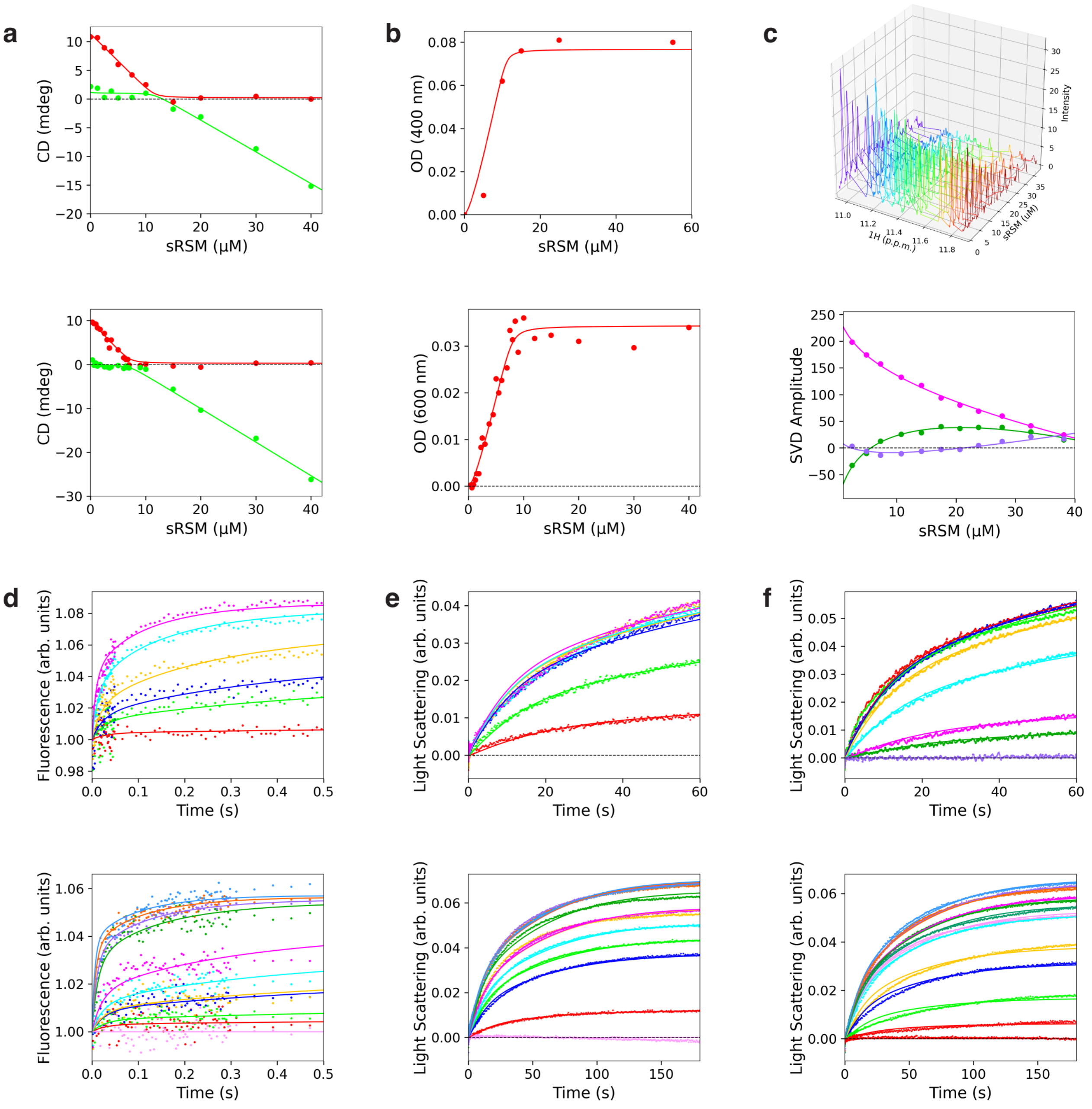

Supplementary Figure 18. Global numerical analysis of sRSM-G4 assembly. (**a**) Circular dichroism (CD) spectra were recorded during the titration of 10 μM G4 with a range of 0 to 40 μM sRSM, each spectrum is an average of four replicated measurements. The CD signal readings at two specific wavelengths, 198 nm and 265 nm, were utilized in the global numerical analysis. (**b**) The optical density recorded at 400 or 600 nm during the titration of 10 μM G4 with a range of 0 to 80 or 0 to 55 μM sRSM. 10 μM G4 was pre-mixed with 5 μM sRSM (upper experiment) and then titrated. Each data point in the bottom experiment represents the average of triplicate measurements, statistics are shown in **Fig. 6c**. (**c**) Input NMR spectra (upper graph) and SVD amplitude vectors (lower graph) dependence on the concentration of 0 to 40 μM sRSM measured with 50 μM G4. (**d**) The stopped-flow fluorescence (excitation 295 nm, emission > 320 nm) traces recorded upon mixing 2.5 μM G4 with 0 – 20 μM RSM. (**e**) The stopped-flow light scattering traces recorded upon rapid mixing 2.5 μM G4 with 0 – 20 μM sRSM and (**f**) 2.5 μM sRSM with 0 – 20 μM G4. Each stopped-flow trace represents the average of 3 to 4 replicates. The solid lines represent the best global fit to the kinetic data. Source data are provided as a Source Data file.

Supplementary Figure 19

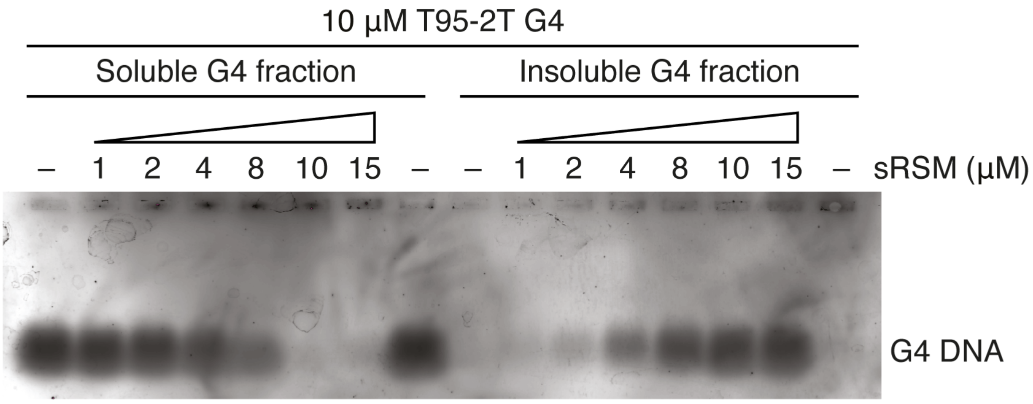

Supplementary Figure 19. Spin-down assay validating the proposed model. Sybr Gold-stained soluble and insoluble fractions of T95-2T G4 separated after incubation with the indicated concentration of sRSM peptide. Prior to analysis on agarose gel sRSM was removed by SDS and proteinase K. Source data are provided as a Source Data file.

Supplementary Figure 20

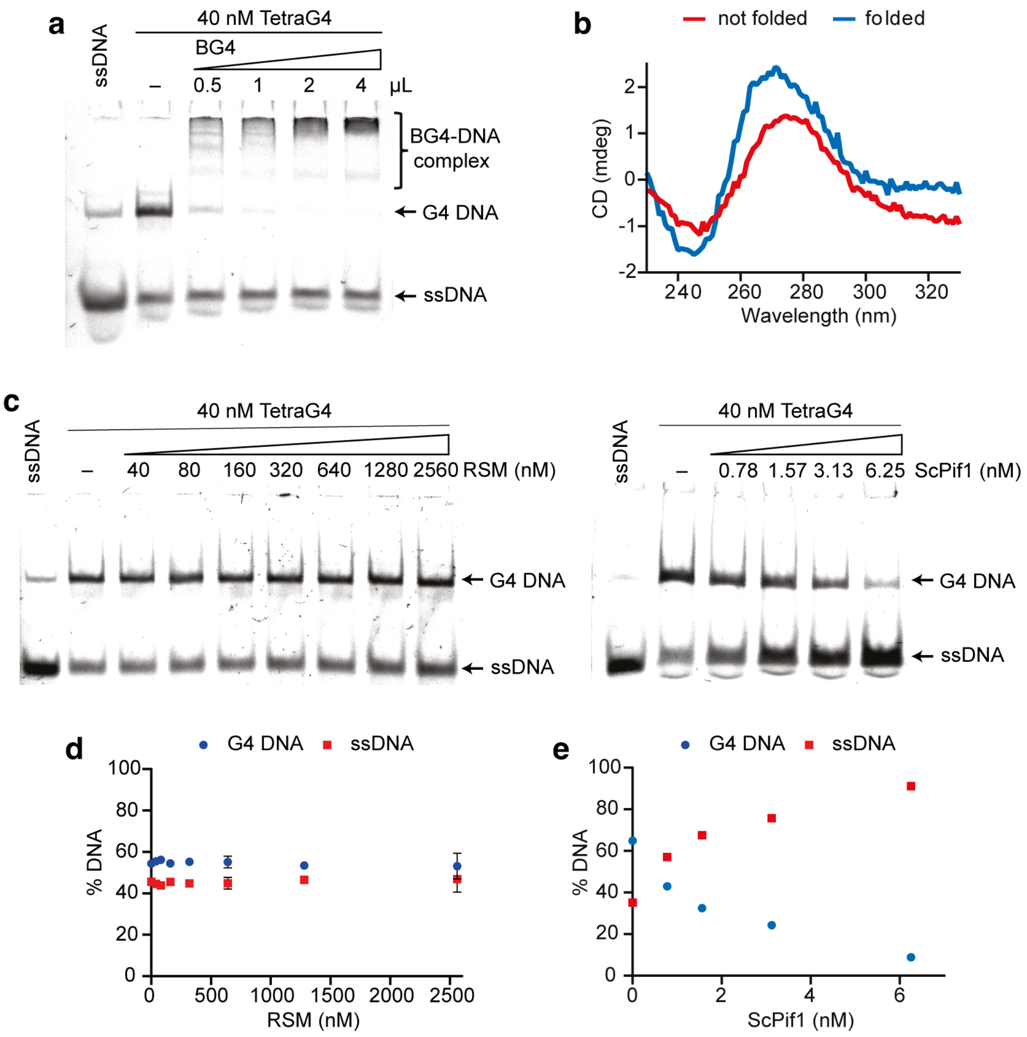

Supplementary Figure 20. Unwinding of a tetramolecular G4 substrate. (**a**) Folding of tetramolecular G4 (TetraG4) was confirmed by EMSA of fluorescently labeled TetraG4 (40 nM) with increasing amount of BG4 antibody. The mixture was separated on PAGE. (**b**) CD spectra of folded (8 µM; blue) and not folded TetraG4 DNA (10 µM; red). (**c**) RSM does not destabilize TetraG4 (left) as opposed to ScPif1 helicase (right) that possesses unwinding activity. Fluorescently labeled TetraG4 (40 nM) was incubated with increasing concentration of RSM or ScPif1 and mixtures were separated on PAGE. (**d**) Quantification of the gel from (c, left); n = 3 independent experiments; data are means ± s.d. (**e**) Quantification of the gel image shown from (c, right); n = 1 independent experiment. Source data are provided as a Source Data file.

Supplementary Figure 21

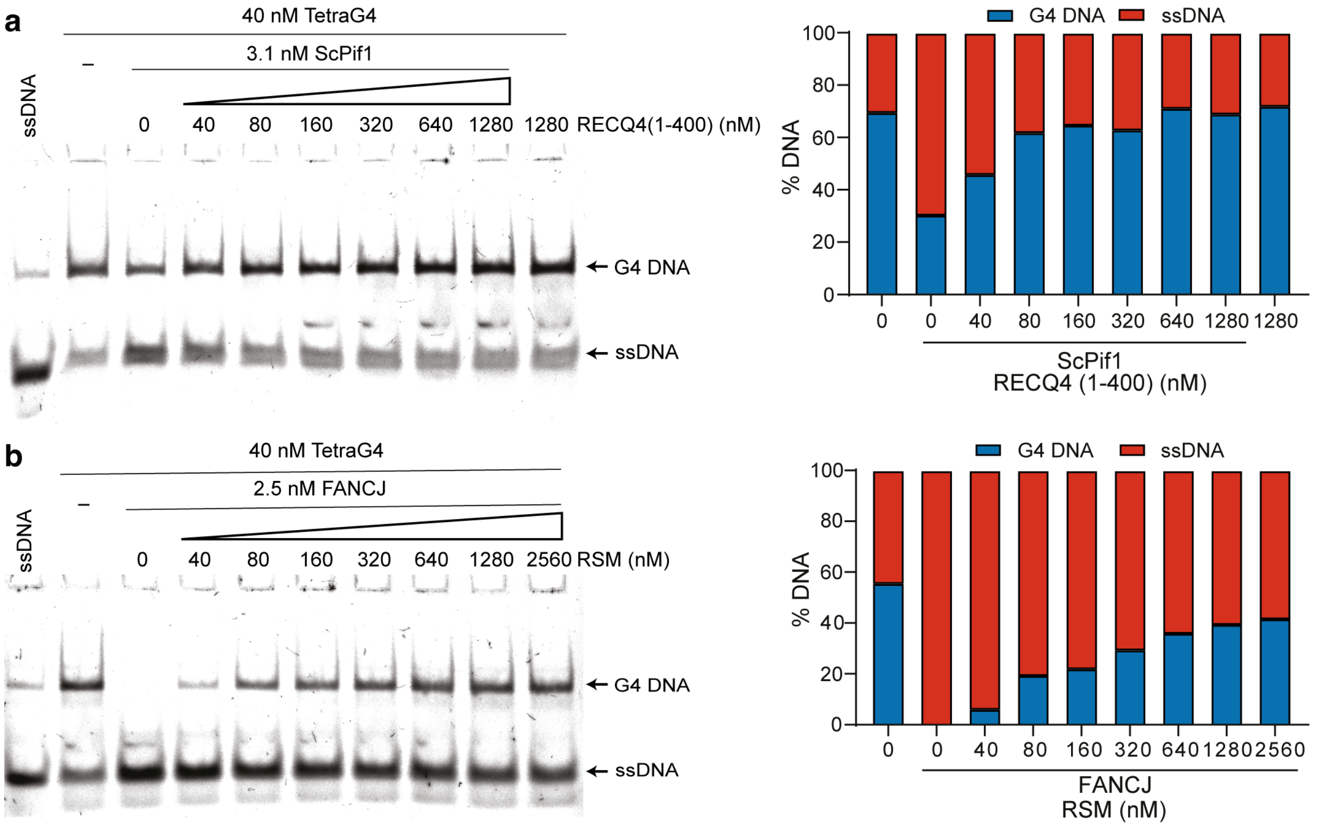

Supplementary Figure 21. RECQ4 fragments hinder G4 processing by other helicases. (**a**) MBP-RECQ4 (1-400) blocks the unwinding activity of ScPif1 helicase (left). Fluorescently labeled tetramolecular G4 (TetraG4; 40 nM) was incubated with scPif1 (3.1 nM) in the absence or presence of increasing concentration of MBP-RECQ4 (1-400). After incubation the reaction mixtures were separated on PAGE and quantified (right); n=1 indepedent experiment. (**b**) RSM (348-388) blocks the unwinding activity of human FANCJ helicase (left). Fluorescently labeled TetraG4 (40 nM) was incubated with FANCJ (2.5 nM) in the absence or presence of increasing concentration of RSM. After incubation the reaction mixtures were separated on PAGE and quantified (right); n=1 indepedent experiment. Source data are provided as a Source Data file.

Supplementary Figure 22

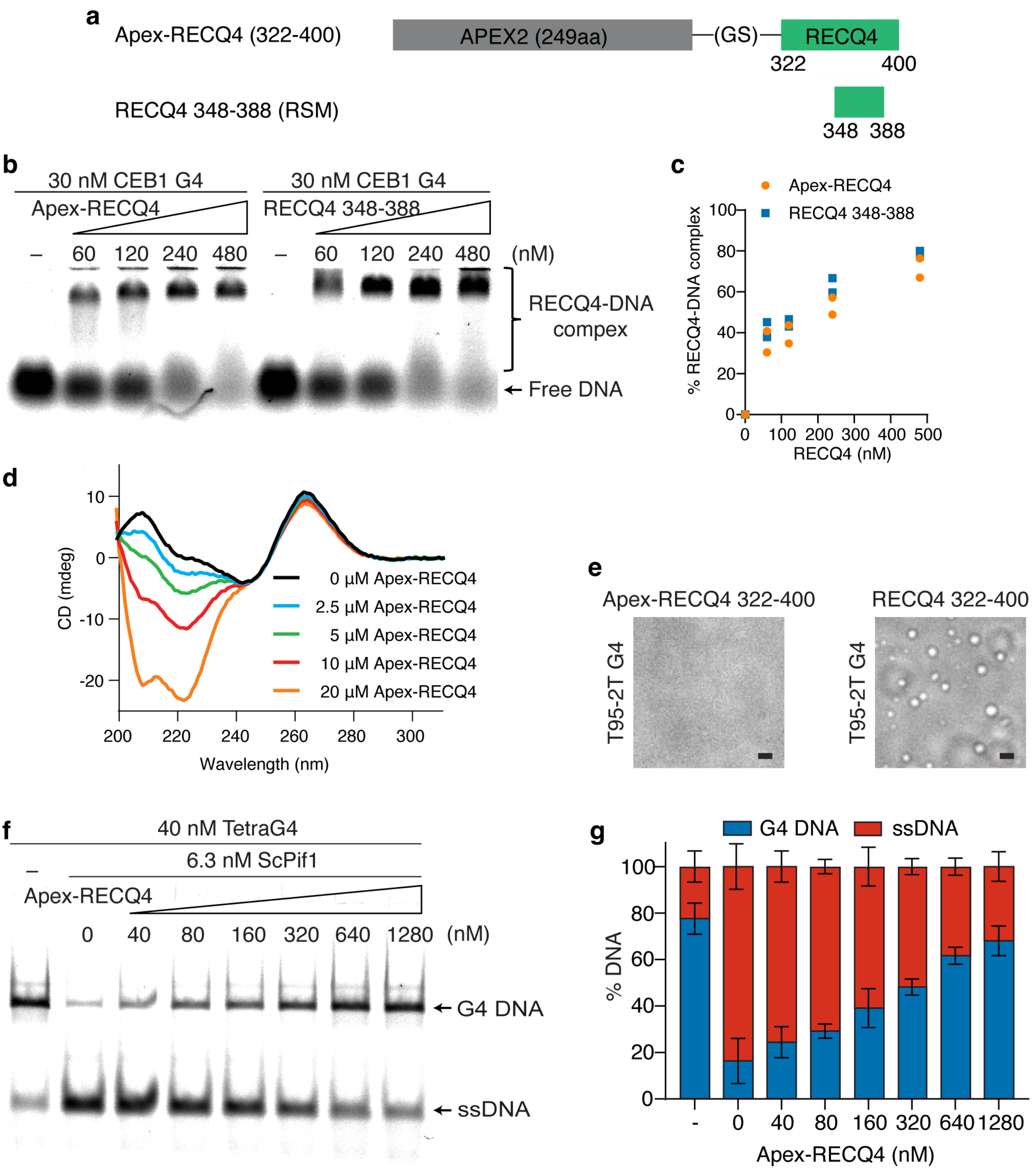

Supplementary Figure 22. RECQ4 (322-400) fusion to APEX2 tag prohibits droplet formation but still binds G4 and hinders G4 processing by other helicases. (**a**) Scheme comparing the Apex-RECQ4 (322-400) to 348-388 (RSM). GS denotes a glycine-serine linker. (**b**) Apex-RECQ4 binds G4 similarly as RSM. Increasing concentration of RECQ4 348-388 (RSM) or Apex-RECQ4 were incubated with fluorescently labeled CEB1 G4 (30 nM). Complexes were resolved on agarose gel. (**c**) Quantification of gel shown in (**b**), n = 2 independent experiments. (**d**) CD spectra of 10 µM parallel G4 (T95-2T) with increasing concentration of Apex-RECQ4. The drop in G4 signal (265 nm) is due to sample dilution. (**e**) Phase separation microscopy of 10 µM Apex-RECQ4 (322-400) or RECQ4 (322-400) mixed with equimolar amount of parallel G4 (T95-2T) and analyzed by DIC microscopy. In both images scale bar = 1 µm. (**f**) Apex-RECQ4 blocks unwinding of tetramolecular G4 (TetraG4) by ScPif1. Fluorescently labeled TetraG4 (40 nM) was incubated with ScPif1 helicase (6.3 nM) in the absence or presence of increasing amounts of Apex-RECQ4. After incubation the reaction mixtures were separated on PAGE. (**g**) Quantification of gel image shown in (**f**), n = 3 independent experiments; data are means ± s.d. Source data are provided as a Source Data file.

Supplementary Figure 23

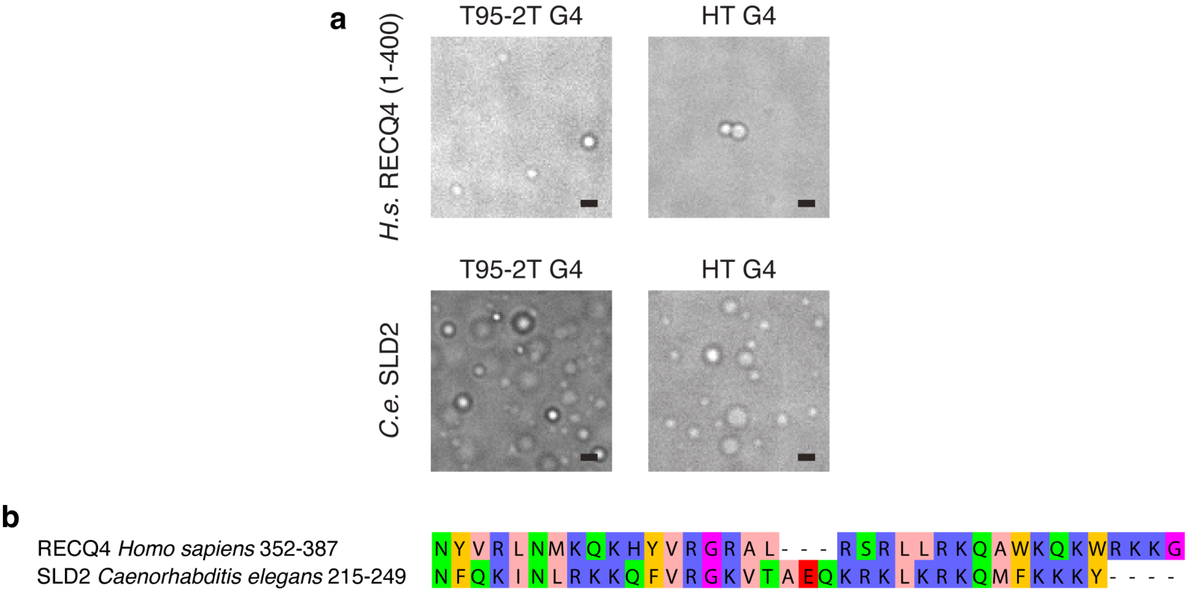

Supplementary Figure 23. Phase separation properties of *H. sapiens* RECQ4 (1-400) and *C. elegans* full-length SLD2 protein. (**a**) Phase separation microscopy of 10 µM *H. sapiens* RECQ4 (1-400) (top) or full length *C. elegans* SLD2 (bottom) were mixed with equimolar amount of parallel G4 (T95-2T) or hybrid G4 (HT) and analyzed by DIC microscopy. In all images scale bar = 1 µm. (**b**) Sequence comparison of RSM-containing region in *H. sapiens* RECQ4 protein and *C. elegans* SLD2 protein.

Supplementary Table 1

Oligonucleotide sequences of DNA substrates used in NMR, FA, EMSA, and DIC
(* indicates FITC or Cy3 position for FA or EMSA)

splayed-arm (Y14)	a) 5′•CAGCGCTCGGTTTT•3′<br>b) 5′•TTTTCCGAGCGCTG•3′
single-stranded (ss10)	*5′•CAGCGCTCGG•3′
single-stranded (ss18)	*5′•GTGATGCGTCAACACTTC•3′
single-stranded (ss20)	*5′•GTTAACCCTAACCCTAAGAT•3′
single-stranded (ss49)	*5′•AGCTACCATGCCTGCACGAATTAAGCAATTCGTAATCATGGTCATAGCT•3′
double-stranded (ds10)	a)*5′•CAGCGCTCGG•3′<br>b) 5′•CCGAGCGCTG•3′
double-stranded (ds40)	a) 5′• CAAGGACGACGATGACAAGTAGGATTTGGATCTACTGGAC•3′<br>b)*5′• GTCCAGTAGATCCAAATCCTACTTGTCATCGTCGTCCTTG•3′
double-stranded (ds49)	a)*5′•AGCTACCATGCCTGCACGAATTAAGCAATTCGTAATCATGGTCATAGCT•3′<br>b) 5′•AGCTATGACCATGATTACGAATTGCTTAATTCGTGCAGGCATGGTAGCT•3′
parallel G4 (T95-2T)	5′•TTGGGTGGGTGGGTGGGT•3′
hybrid G4 (HT)	*5′•TTGGGTTAGGGTTAGGGTTAGGGA•3′
parallel G4 (CEB1)	*5′•AGGGGGGAGGGAGGGTGG•3′
tetramolecular G4	*5′• ACTGTCGTACTTGATATTGGGGC•3′ (4×)

Supplementary Table 2

Chemical shift based secondary structure (SS) prediction of RSM free (as part of RECQ4 322-400)

H = helix E = strand L = coil

	NMR backbone chemical shifts measured						TALOS prediction			
aa	HA	HN	N	CA	CB	C	P_{helix}	P_{strand}	P_{coil}	SS
H348	—	8.270	119.9	—	30.42	—	0.063	0.088	0.849	L
D349	4.618	8.266	121.5	54.39	41.44	176.3	0.173	0.035	0.792	L
R350	4.318	8.322	121.2	56.60	30.66	177.0	0.356	0.069	0.575	L
G351	3.907	8.392	108.8	45.66	—	174.0	0.084	0.011	0.905	L
N352	4.675	8.171	118.6	53.41	38.96	175.0	0.004	0.036	0.960	L
Y353	4.542	8.025	120.6	58.30	38.80	175.8	0.038	0.082	0.880	L
V354	3.987	7.896	121.8	62.84	32.78	175.9	0.065	0.133	0.802	L
R355	4.308	8.153	124.1	56.39	30.78	176.4	0.056	0.144	0.800	L
L356	4.297	8.128	123.0	55.55	42.59	177.1	0.092	0.061	0.848	L
N357	4.671	8.335	119.0	53.43	38.80	175.4	0.053	0.040	0.906	L
M358	4.429	8.168	120.5	55.97	32.92	176.4	0.092	0.050	0.858	L
K359	4.273	8.199	121.6	56.81	32.85	176.7	0.079	0.049	0.871	L
Q360	4.282	8.157	120.7	56.07	29.64	176.0	0.067	0.056	0.877	L
K361	—	8.213	122.1	56.67	33.10	176.3	0.066	0.046	0.888	L
H362	—	—	—	56.00	30.70	174.7	0.043	0.059	0.898	L
Y363	4.577	8.081	122.1	57.95	39.09	175.5	0.024	0.204	0.772	L
V364	4.027	8.019	122.8	62.39	33.05	175.8	0.015	0.384	0.601	L
R365	—	8.292	124.9	56.58	30.91	176.8	0.039	0.103	0.858	L
G366	3.925	8.353	109.9	45.54	—	174.1	0.048	0.034	0.919	L
R367	4.288	8.177	120.3	56.53	30.99	176.3	0.204	0.057	0.739	L
A368	4.299	8.271	124.8	52.78	19.15	177.9	0.193	0.073	0.734	L
L369	4.296	8.091	121.6	55.67	42.38	177.7	0.296	0.069	0.634	L
R370	4.305	8.216	121.2	56.74	30.61	176.7	0.428	0.054	0.517	L
S371	4.387	8.156	116.1	58.96	63.77	175.0	0.261	0.017	0.722	L
R372	4.293	8.250	122.9	56.94	30.77	176.5	0.325	0.037	0.638	L
L373	4.301	8.058	122.1	55.66	42.31	177.4	0.458	0.079	0.463	L
L374	4.331	8.026	122.6	55.50	42.34	177.5	0.353	0.067	0.580	L
R375	4.295	8.101	121.5	56.44	30.85	176.5	0.325	0.032	0.642	L
K376	4.229	8.175	122.0	56.90	33.01	176.8	0.299	0.042	0.660	L
Q377	4.217	8.252	120.9	56.18	29.36	176.0	0.276	0.037	0.687	L
A378	4.257	8.189	124.8	53.02	19.18	177.7	0.156	0.032	0.812	L
W379	4.595	7.943	119.8	57.66	29.49	176.4	0.320	0.073	0.607	L
K380	4.099	7.801	122.1	56.75	33.05	176.4	0.305	0.071	0.624	L
Q381	4.129	7.954	120.2	56.26	31.56	176.1	0.093	0.055	0.852	L
K382	4.173	8.067	121.7	56.93	32.92	176.3	0.028	0.051	0.921	L
W383	4.675	7.903	120.9	57.10	29.74	176.1	0.037	0.066	0.897	L
R384	4.243	7.939	122.4	56.10	31.10	175.9	0.051	0.077	0.872	L
K385	4.203	8.127	122.4	56.45	33.30	176.5	0.047	0.054	0.899	L
K386	4.233	8.253	122.7	56.95	33.06	177.0	0.038	0.042	0.920	L
G387	3.980	8.360	110.4	45.43	—	174.2	0.145	0.022	0.832	L
E388	—	8.134	120.5	56.69	30.67	176.4	0.333	0.333	0.333	L

Supplementary Table 3

Chemical shift based secondary structure (SS) prediction RSM bound to ds10 DNA

H = helix E = strand L = coil

	NMR backbone chemical shifts measured						TALOS prediction			
aa	HA	HN	N	CA	CB	C	P_{helix}	P_{strand}	P_{coil}	SS
H348	4.587	8.355	117.6	55.22	29.77	174.4	0.333	0.333	0.333	L
D349	4.542	8.293	121.2	53.92	41.44	176.2	0.060	0.066	0.874	L
R350	4.247	8.374	121.6	56.11	30.61	176.9	0.042	0.049	0.910	L
G351	3.851	8.425	108.8	45.19	–	173.9	0.020	0.045	0.936	L
N352	4.602	8.186	118.5	52.87	38.91	174.8	0.011	0.052	0.937	L
Y353	4.437	8.006	120.5	57.91	38.82	175.6	0.012	0.129	0.860	L
V354	3.889	7.899	122.2	62.36	32.75	175.8	0.023	0.265	0.712	L
R355	4.206	8.171	124.2	55.86	30.80	176.3	0.011	0.286	0.703	L
L356	4.228	8.175	123.0	55.07	42.68	177.0	0.022	0.122	0.856	L
N357	4.608	8.352	118.7	52.9	38.71	175.2	0.015	0.085	0.900	L
M358	4.371	8.200	120.2	55.47	32.83	176.4	0.023	0.073	0.904	L
K359	4.213	8.230	121.5	56.39	32.86	176.6	0.026	0.062	0.912	L
Q360	4.215	8.209	120.6	55.54	29.66	175.9	0.026	0.069	0.905	L
K361	4.146	8.261	122.1	56.17	33.15	176.0	0.012	0.102	0.886	L
H362	4.600	8.343	119.4	55.04	29.98	174.1	0.022	0.252	0.726	L
Y363	4.502	8.199	122.2	57.48	39.18	175.3	0.000	0.637	0.363	L
V364	3.981	8.125	123.2	61.85	33.2	175.6	0.000	0.654	0.346	L
R365	4.213	8.400	125.1	56.11	30.94	176.8	0.012	0.133	0.855	L
G366	3.927	8.483	110.0	45.14	–	174.2	0.008	0.054	0.938	L
R367	4.220	8.180	120.9	56.18	31.03	176.3	0.082	0.069	0.849	L
A368	4.240	8.368	124.7	52.46	19.14	178.0	0.058	0.112	0.830	L
L369	4.224	8.154	121.4	55.41	42.22	177.7	0.094	0.118	0.788	L
R370	4.227	8.282	120.9	56.5	30.71	176.7	0.149	0.063	0.788	L
S371	4.331	8.199	115.8	58.68	63.79	175.1	0.083	0.027	0.890	L
R372	4.215	8.298	122.9	56.75	30.66	176.8	0.144	0.067	0.789	L
L373	4.223	8.104	121.6	55.5	42.13	177.6	0.275	0.111	0.614	L
L374	4.240	8.021	121.8	55.25	42.26	177.7	0.148	0.070	0.782	L
R375	4.219	8.111	121.2	56.27	30.74	176.6	0.110	0.046	0.845	L
K376	4.146	8.186	121.6	56.61	32.9	176.9	0.085	0.048	0.867	L
Q377	4.145	8.245	120.3	55.87	29.24	176.1	0.070	0.047	0.883	L
A378	4.173	8.190	124.2	52.79	19.14	177.9	0.061	0.061	0.878	L
W379	4.457	7.940	119.6	57.4	29.49	176.4	0.122	0.111	0.767	L
K380	3.967	7.765	121.7	56.42	33.03	176.4	0.079	0.068	0.853	L
Q381	4.043	7.936	119.8	55.73	29.21	176.0	0.024	0.049	0.927	L
K382	4.084	8.035	121.4	56.46	32.87	176.2	0.015	0.053	0.931	L
W383	4.537	7.878	121.0	56.65	29.64	175.9	0.020	0.100	0.880	L
R384	4.159	7.841	122.6	55.44	31.24	175.6	0.023	0.124	0.853	L
K385	4.120	8.198	123.1	55.84	33.29	176.3	0.015	0.090	0.895	L
K386	4.181	8.358	123.3	56.52	33.04	177.0	0.016	0.081	0.904	L
G387	3.901	8.452	111.7	44.87	–	173.1	0.055	0.063	0.882	L
E388	–	7.824	125.5	57.44	–	–	0.333	0.333	0.333	L

Supplementary Table 4

Chemical shift based secondary structure (SS) prediction of RSM bound to RPA32C

H = helix **E = strand** **L = coil**

	NMR backbone chemical shifts measured						TALOS prediction			
aa	HA	HN	N	CA	CB	C	P_{helix}	P_{strand}	P_{coil}	SS
H348	4.539	8.302	117.8	55.64	29.63	174.5	0.333	0.333	0.333	L
D349	4.497	8.240	121.2	54.31	41.29	176.3	0.287	0.007	0.706	L
R350	4.211	8.323	121.5	56.50	30.47	176.9	0.081	0.016	0.904	L
G351	3.804	8.376	108.8	45.56	0.000	174.0	0.023	0.038	0.939	L
N352	4.564	8.141	118.5	53.32	38.74	174.9	0.034	0.030	0.935	L
Y353	4.412	7.992	120.5	58.34	38.64	175.7	0.044	0.060	0.896	L
V354	3.849	7.879	121.9	62.86	32.67	175.9	0.108	0.048	0.844	L
R355	4.160	8.118	124.0	56.37	30.63	176.4	0.070	0.057	0.873	L
L356	4.177	8.109	122.8	55.47	42.48	177.1	0.210	0.051	0.739	L
N357	4.557	8.307	118.8	53.33	38.55	175.4	0.109	0.025	0.865	L
M358	4.288	8.150	120.1	56.12	32.66	176.5	0.250	0.050	0.700	L
K359	4.142	8.152	121.3	56.97	32.66	176.9	0.267	0.039	0.694	L
Q360	4.159	8.128	120.4	56.17	29.42	176.2	0.147	0.023	0.830	L
K361	4.074	8.198	121.8	56.90	32.89	176.5	0.167	0.036	0.797	L
H362	4.522	8.231	119.1	55.86	29.85	174.7	0.094	0.035	0.871	L
Y363	4.424	8.119	122.0	58.31	38.85	175.7	0.058	0.057	0.885	L
V364	3.852	8.033	122.6	62.82	32.81	176.0	0.079	0.054	0.867	L
R365	4.122	8.259	124.3	56.90	30.66	177.1	0.104	0.017	0.878	L
G366	3.847	8.332	109.4	45.83	–	174.6	0.370	0.023	0.606	L
R367	4.070	8.074	121.2	57.38	30.58	177.0	0.751	0.018	0.231	H
A368	4.107	8.223	123.9	53.56	18.65	178.7	0.923	0.006	0.071	H
L369	4.076	7.984	120.8	56.50	41.90	177.9	0.956	0.000	0.044	H
R370	4.012	8.105	119.9	57.91	30.23	177.4	0.966	0.000	0.034	H
S371	4.198	8.050	114.8	60.02	63.33	175.7	0.953	0.000	0.047	H
R372	4.013	8.079	122.5	58.32	30.33	177.5	0.952	0.000	0.048	H
L373	4.104	8.054	120.5	56.64	41.73	178.5	0.977	0.000	0.023	H
L374	4.116	7.926	121.1	56.52	41.85	178.6	0.979	0.000	0.021	H
R375	4.046	7.934	120.7	57.72	30.31	177.3	0.950	0.000	0.050	H
K376	4.029	8.138	121.0	57.99	32.52	177.3	0.937	0.000	0.063	H
Q377	4.027	8.103	119.3	57.06	28.84	176.8	0.915	0.000	0.085	H
A378	4.078	7.990	123.2	53.77	18.59	178.9	0.895	0.000	0.105	H
W379	4.398	7.920	119.4	58.70	29.12	177.2	0.924	0.005	0.070	H
K380	3.957	7.857	120.4	57.54	32.69	177.4	0.830	0.007	0.163	H
Q381	4.001	7.834	118.7	56.79	28.87	176.6	0.418	0.001	0.581	L
K382	4.001	7.831	120.4	57.26	32.64	176.7	0.159	0.007	0.834	L
W383	4.465	7.795	120.2	57.28	29.56	176.0	0.107	0.039	0.854	L
R384	4.134	7.722	121.9	56.00	31.01	175.8	0.100	0.035	0.865	L
K385	4.105	8.160	122.9	56.26	33.05	176.4	0.039	0.028	0.933	L
K386	4.144	8.271	123.0	56.85	32.86	177.0	0.049	0.026	0.925	L
G387	3.843	8.388	111.5	45.25	–	173.1	0.256	0.017	0.727	L
E388	4.055	7.762	125.4	57.80	31.09	–	0.333	0.333	0.333	L

Supplementary Table 5

Primer sequences for the preparation of constructs used in this study
Lowercase underlined denotes overhang
Uppercase underlined denotes stop codon
Lowercase bold denotes restriction enzyme sequence

Construct	Forward primer	Reverse primer
RPA70$_{(1-120)}$	aaa**ccatgg**TCGGCCAGCTGAGCGAGG	tta**ggatcc**TTATTCATTATAGGGCACTGG
RPA32$_{(172-270)}$	aaa**ccatgg**CCAACAGCCAGCCCTCAGC	tta**ggatcc**TTATTCTGCATCTGTGGATTTAAAATG
RECQ4$_{(1-400)}$	aaa**tcatga**TGGAGCGGCTGCGGGACGTG	tta**ggatcc**TCACTTGGTTGTGACTGTGGCA
RECQ4$_{(1-150)}$	tta**ccatgg**AGCGGCTGCGGGACGTGCGGGAG	tta**ggatcc**TCAGGAGGGGACAGGCCCTGTACCTGGGGGC
RECQ4$_{(150-315)}$	aaa**ccatgg**GCTCCTTTGCAGAAAAAGTCAGTGATG	tta**ggatcc**TTACGATGGGCTGCTGCAGGGCTGA
RECQ4$_{(322-400)}$	tta**ccatgg**GACTCAGCCCCTCCAGTCA	tta**ggatcc**TCACTTGGTTGTGACTGTGGCA
RECQ4$_{(348-388)}$	tta**ccatgg**CACATGACAGGGGCAATTAC	tta**ggatcc**TCACTCCCCTTTCTTCCGCCA
RECQ4$_{(348-388)}$ 5E mutant	CGTAGCAGGCTCCTCGAGGAGCAGGCATGGGAGCAGGAGTGGGAGAAGAAAGGGGAG	CTCCCCTTTCTTCTCCCACTCCTGCTCCCATGCCTGCTCCTCGAGGAGCCTGCTACG
RECQ4$_{(348-388)}$ W379A/W383A mutant	CGCAAGCAGGCAGCGAAGCAGAAGGCGCGGAAGAAA	TTTCTTCCGCGCCTTCTGCTTCGCTGCCTGCTTGCG
C. elegans SLD2 full-length	tta**ccatgg**AAGAGTGGAAAACGTCT	tta**ggatcc**TCAGTACTTCTTCTTGAACAT

Supplementary Table 6

Buffer and temperature of NMR experiments
(all buffers contained 10% D_2O for the lock)

NMR data displayed in	Buffer composition and temperature in Kelvin
**Fig. 2a,b**	50 mM NaPO4, pH 6.0, 100 mM NaCl, 1 mM TCEP, 1 mM d6-EDTA, 298 K
**Fig. 3a,b**	50 mM NaPO4, pH 6.0, 150 mM NaCl, 1 mM TCEP, 1 mM d6-EDTA, 5% d8-glycerol, 298 K
**Fig. 4a,b**	50 mM NaPO4, pH 6.0, 150 mM NaCl, 1 mM TCEP, 1 mM d6-EDTA, 5% d8-glycerol, 298 K
**Fig. 5a,b**	25 mM KPO4, pH 6.5, 70 mM KCl, 298 K
**Supplementary Fig. 3b**	25 mM Hepes, pH 7.0, 120 mM NaCl, 2 mM BME, 0.25% d8-glycerol, 293 K
**Supplementary Fig. 3c,d**	30 mM NaPO4, pH 6.0, 100 mM NaCl, 1 mM TCEP, 298 K
**Supplementary Fig. 4a**	50 mM NaPO4, pH 6.0, 100 mM NaCl, 1 mM TCEP, 1 mM d6-EDTA, 298 K
**Supplementary Fig. 5c**	25 mM KPO4, pH 6.5, 70 mM KCl, 80 mM NaCl, 5% d8-glycerol, 298 K
**Supplementary Fig. 6**	50 mM NaPO4, pH 6.0, 100 mM NaCl, 1 mM TCEP, 298 K
**Supplementary Fig. 7a**	20mM NaPO4, pH 6.0, 100mM NaCl, 2 mM BME, 278 K
**Supplementary Fig. 7d**	50 mM NaPO4, pH 6.0, 150 mM NaCl, 1 mM d6-EDTA, 5% d8-glycerol, 298 K
**Supplementary Fig. 8**	20 mM NaPO4, pH 6.0, 300 mM NaCl, 1 mM d6-EDTA, 5% d8-glycerol, 288 K
**Supplementary Fig. 9**	50 mM NaPO4, pH 6.0, 150 mM NaCl, 1 mM TCEP, 1 mM d6-EDTA, 5% d8-glycerol, 298 K
**Supplementary Fig. 10**	50 mM NaPO4, pH 6.0, 150 mM NaCl, 1 mM TCEP, 1 mM d6-EDTA, 5% d8-glycerol, 298 K
**Supplementary Fig. 13a,c**	20 mM KPO4, pH 6.5, 70 mM KCl, 1 mM d6-EDTA, 298 K
**Supplementary Fig. 14e**	20 mM KPO4, pH 6.5, 70 mM KCl, 1 mM d6-EDTA, 298 K
**Supplementary Fig. 15**	25 mM KPO4, pH 6.5, 70 mM KCl, 298 K

Supplementary Table 7

The kinetic parameters and scaling factors obtained by global fit
The best-fit estimates of the kinetic parameters and the signal scaling factors were obtained by nonlinear regression based on numerical integration of the rate equations derived from the input kinetic model (**Fig. 6f**). The standard error (± s.e.) was calculated from the covariance matrix during nonlinear regression.

**Parameter**		**best-fit ± s.e.**	Scaling factors (replicate 1)	best-fit ± s.e.	Scaling factors (replicate 2)	best-fit ± s.e.
$\boldsymbol{k_1}$	**µM^{-1}.s^{-1}**	**5.4 ± 0.2**	a_{265}	0.98 ± 0.01	a_{265}	1.37 ± 0.01
			a_{196}	1.16 ± 0.06	a_{196}	0.21 ± 0.03
$\boldsymbol{k_{-1}}$	**s^{-1}**	**17.4 ± 0.9**	b_{196}	0.55 ± 0.01	b_{196}	0.76 ± 0.01
			a_{FI}	0.0224 ± 0.0005	a_{FI}	0.0179 ± 0.0003
$\boldsymbol{k_2}$	**µM^{-1}.s^{-1}**	**1.9 ± 0.1**	b_{FI}	0.086 ± 0.001	b_{FI}	0.055 ± 0.001
			a_{OD}	0.0075 ± 0.0003	a_{OD}	0.0051 ± 0.0002
$\boldsymbol{k_{-2}}$	**s^{-1}**	**0.19 ± 0.01**	a_{LS} (G4 fixed)	0.030 ± 0.001	a_{LS} (G4 fixed)	0.042 ± 0.002
			a_{LS} (sRSM fixed)	0.043 ± 0.002	a_{LS} (sRSM fixed)	0.049 ± 0.002
$\boldsymbol{k_3}$	**µM^{-1}.s^{-1}**	**0.21 ± 0.01**	$b_{n=3}$	3.3 ± 0.4	$b_{n=3}$	3.3 ± 0.4
			$b_{n=4}$	3.3 ± 1.1	$b_{n=4}$	3.3 ± 1.1
$\boldsymbol{k_{-3}}$	**s^{-1}**	**0.044 ± 0.004**	$b_{n=5}$	6.2 ± 2.8	$b_{n=5}$	6.2 ± 2.8
			$b_{n=6}$	13.6 ± 2.3	$b_{n=6}$	13.6 ± 2.3

Supplementary References

1. Erdos, G., Pajkos, M. & Dosztanyi, Z. IUPred3: prediction of protein disorder enhanced with unambiguous experimental annotation and visualization of evolutionary conservation. *Nucleic Acids Res.* **49**, W297-W303, (2021).
2. Jumper, J. *et al.* Highly accurate protein structure prediction with AlphaFold. *Nature* **596**, 583-589, (2021).
3. Edgar, R. C. MUSCLE: multiple sequence alignment with high accuracy and high throughput. *Nucleic Acids Res.* **32**, 1792-1797, (2004).
4. Ashkenazy, H. *et al.* ConSurf 2016: an improved methodology to estimate and visualize evolutionary conservation in macromolecules. *Nucleic Acids Res.* **44**, W344-350, (2016).
5. Bolognesi, B. *et al.* A Concentration-Dependent Liquid Phase Separation Can Cause Toxicity upon Increased Protein Expression. *Cell Rep* **16**, 222-231, (2016).
